# Nonlethal deleterious mutation–induced stress accelerates bacterial aging

**DOI:** 10.1073/pnas.2316271121

**Published:** 2024-05-06

**Authors:** Maryam Kohram, Amy E. Sanderson, Alicia Loui, Peyton V. Thompson, Harsh Vashistha, Aseel Shomar, Zoltán N. Oltvai, Hanna Salman

**Affiliations:** ^a^Department of Physics and Astronomy, University of Pittsburgh, Pittsburgh, PA 15260; ^b^Department of Pathology, University of Pittsburgh, Pittsburgh, PA 15260; ^c^Department of Chemical Engineering, Technion–Israel Institute of Technology, Haifa 32000, Israel; ^d^Department of Computational and Systems Biology, University of Pittsburgh, Pittsburgh, PA 15260; ^e^Department of Pathology and Laboratory Medicine, University of Rochester, Rochester, NY 14627

**Keywords:** adaptation, resilience, stress, replicative lifespan, metabolism

## Abstract

Random mutagenesis, including when it leads to loss of gene function, is a key mechanism enabling microorganisms’ long-term adaptation to new environments. However, loss-of-function mutations are often deleterious, triggering, in turn, cellular stress and complex homeostatic stress responses, called “allostasis,” to promote cell survival. Here, we characterize the differential impacts of 65 nonlethal, deleterious single-gene deletions on *Escherichia coli* growth in three different growth environments. Further assessments of select mutants, namely, those bearing single adenosine triphosphate (ATP) synthase subunit deletions, reveal that mutants display reorganized transcriptome profiles that reflect both the environment and the specific gene deletion. We also find that ATP synthase α-subunit deleted (*ΔatpA*) cells exhibit elevated metabolic rates while having slower growth compared to wild-type (wt) *E. coli* cells. At the single-cell level, compared to wt cells, individual *ΔatpA* cells display near normal proliferation profiles but enter a postreplicative state earlier and exhibit a distinct senescence phenotype. These results highlight the complex interplay between genomic diversity, adaptation, and stress response and uncover an “aging cost” to individual bacterial cells for maintaining population-level resilience to environmental and genetic stress; they also suggest potential bacteriostatic antibiotic targets and -as select human genetic diseases display highly similar phenotypes, - a bacterial origin of some human diseases.

## Significance

Genomic heterogeneity is pervasive in living organisms, from bacterial ecosystems to human tissues, allowing both adaptability and/or reestablishment of homeostasis in the proper context. In nature, mutations that cause loss of gene function are a major contributor to bacterial ecosystems’ adaptation to new environments. However, how bacteria handle random, nonlethal, but deleterious loss-of-gene-function mutations is only partially understood. In this study, we show both at population- and single-cell levels that single-gene-deleted *Escherichia coli* mutants activate a homeostatic stress response, i.e., allostasis, which reflects both the nature of the environment and the deleted gene function. Stress and allostasis, however, also lead to early onset of aging in mutant bacteria, thus representing a functional cost for maintaining population-level resilience to environmental challenges.

Adaptation of cells and microorganisms to changes in their environment occurs through both genetic and nongenetic changes. For example, microbial cells may temporarily reorganize their metabolic network activity based on the nature of the new environment (1–3) and by their position within a colony (4). Global reorganization of bacterial physiology can also occur in response to antibiotic challenges. Longer-term adaptation to such challenges, on the other hand, can involve genetic and epigenetic mechanisms (5–7). Random mutagenesis within a bacterial colony enables the coexistence of genetically diverse cells upon which natural selection can act. These include both mutations that improve e.g., enzymatic activities and loss-of-expression/function variants that are beneficial for survival and/or proliferation in a new environment (8–10). However, another unavoidable outcome of random mutagenesis is the emergence of deleterious variants that affect cell growth negatively. In its extreme form, such as the inactivation of essential genes, this leads to cell death and the elimination of these mutations from the pool of genetic variants. In other cases, deleterious mutations persist in the population despite being detrimental to population growth. This raises important questions in evolution: How do such deleterious mutation-bearing microbial cells survive and continue to exist in the colony for an extended time? What are the consequences of such deleterious mutations to cells’ physiology in different environments?

It has long been known that the effect of a mutation depends on the environment the cells are subject to, i.e., the mutation might negatively affect the fitness of cells in some environments but have a neutral or even positive effect in other environments. Another postulate is that the presence of deleterious mutations may not manifest until late in the life cycle of a cell, or that its deleterious effects gradually accumulate as the cell ages, which in turn would lead to the preservation of that mutation in the population. In this case, mutant cells could grow and function similarly to their wild-type (wt) counterpart until homeostatic buffering of the deleterious effect of these mutations is exhausted, leading to accelerated aging and/or cell death.

In this study, we examine the effects of select, nonlethal but deleterious single-gene deletions on the proliferation and physiology of *Escherichia coli* cells under different environmental conditions to uncover how such mutations can persist within a bacterial population. We first show that the effect of such loss-of-gene-function mutations on population growth is indeed context-dependent. To better understand this phenomenon, we focus on single subunit deletion mutants of the ATP synthase. This choice is motivated by the fact that the *E. coli* ATP synthase, which is an F-type synthase, is a well-characterized enzyme complex that plays a central role in bacterial physiology. We find that the growth patterns of the various subunit deletion mutants in different environments vary significantly. This variation is also evident in the gene expression profiles of the different mutant strains that entail activation of homeostatic stress responses, and also reflect the various tested environments. We further examine, at the functional level, a representative ATP synthase mutant (*ΔatpA*), in which the α-subunit is deleted. Despite its reduced growth rate, we find that *ΔatpA* cells have substantially increased oxygen consumption and acidification rates relative to the wt strain. This, in turn, is accompanied by a significant increase in the expression of genes used during anaerobic respiration.

Finally, we compare the growth dynamics of *ΔatpA* mutant and wt *E. coli* cells at the single-cell level. We find that individual *ΔatpA* cells display only a slight alteration in their division dynamics with a change seen only in the last replicative cycle before proliferation arrest. However, mutant cells display accelerated aging, especially in nutrient-limited medium, and enter a postreplicative state earlier than wt cells do with an altered senescence phenotype. Thus, random deleterious mutation-induced homeostatic stress response appears to represent an aging/functional cost for bacterial cells.

## Results

### Single-Gene-Deletion Mutant *E. coli* Strains Display Reduced Growth Efficiency in an Environment-Dependent Manner.

**Fig. 1.**
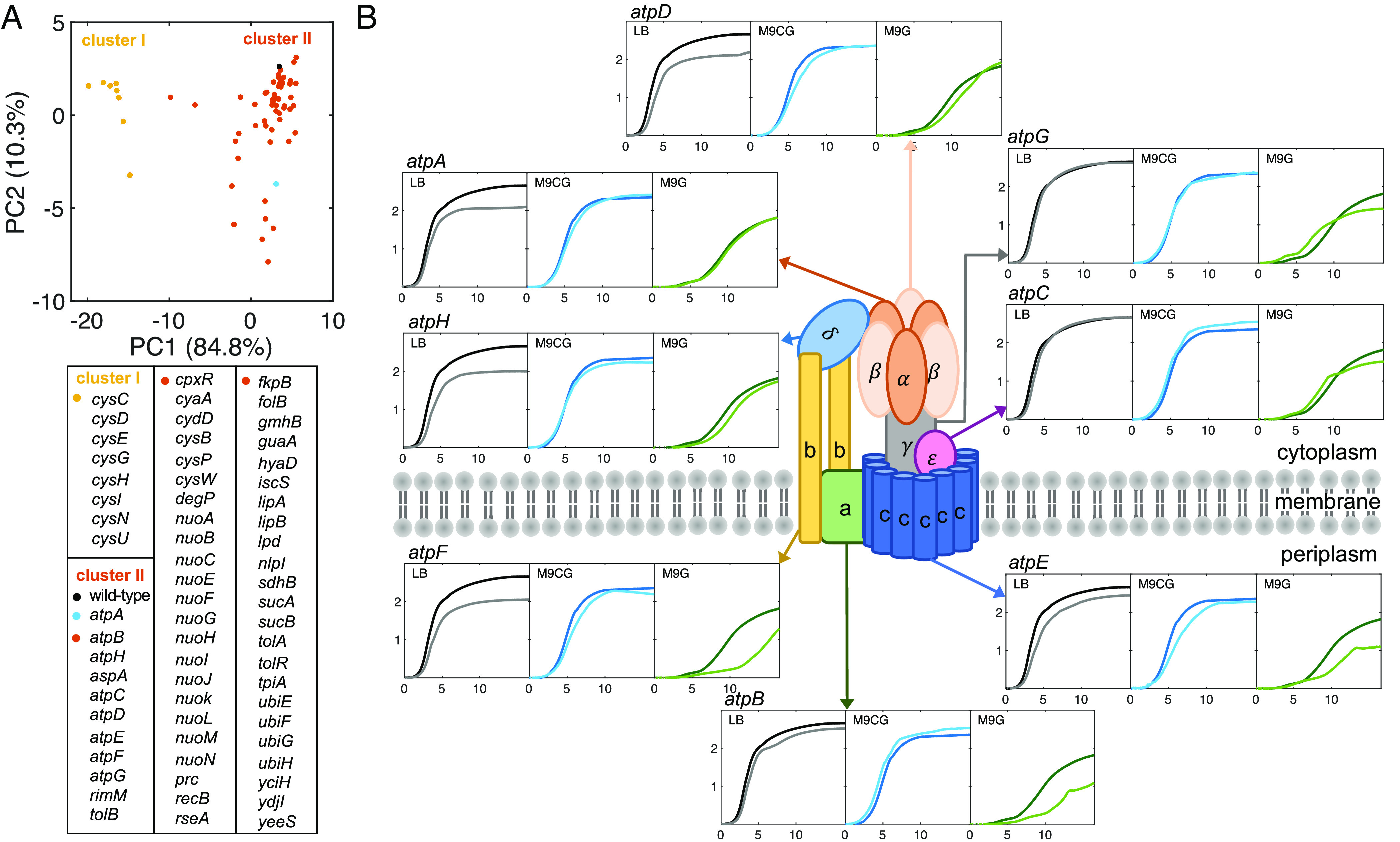
Growth characteristics of *E. coli* single-gene deletion mutants in different nutrient conditions. (*A*) PCA of the growth parameters (*SI Appendix*, Fig. S2*B*) of wt *E. coli* and 65 of its isogenic single-gene deletion mutants identifies two clusters (I [yellow] and II [orange]) based on the first two principal components (PC1 and PC2). Cluster I encompasses deletions from the cysteine metabolism pathway (*SI Appendix*, Fig. S3). wt (black dot) and *ΔatpA* (blue dot) are in cluster II together with the majority of the other single gene deletion strains, indicating similarities in their population growth profiles. (*B*) The ATP synthase complex, embedded in the bacterial inner (cytoplasmic) membrane, comprises two modules (F_0_ and F_1_). Growth profiles of wt (darker lines) and isogenic mutants with individual subunits deleted (lighter lines) are compared in three nutrient media: LB (black), M9CG (blue), and M9G (green). The *x*-axis of all graphs represents time (hr), and *y*-axis is the measured concentration ($ln(\frac{N}{N_{0}})$, where N is ${\mathrm{OD}}_{600}$ measurement and $N_{0}$ is the initial ${\mathrm{OD}}_{600}$ value).

To explore the effects of random deleterious inactivating mutations on bacterial physiology, we first tested the growth of wt *E. coli* K-12 strain BW25113 and 65 of its isogenic single-gene-deletion mutant strains (listed in Fig. 1*A* and *SI Appendix*, Table S1). In a previous study, these mutants displayed the most severe growth defects under single substrate-limited growth conditions in a soft-agar colony assay (11). Here, we tested the growth of these mutants in liquid microbatch cultures of three different growth media as a proxy for different environments: in a rich medium (Luria broth [LB]) that results in rapid growth (∼ 30 min doubling time at 32 °C for wt strain), in a medium (M9CG) resulting in average growth (∼31 min doubling time at 32 °C for wt strain), and in a minimal medium (M9G) resulting in slow growth (∼100 min doubling time at 32 °C for wt strain) (see *Materials and Methods* for media composition). Note that during liquid culture growth, bacteria continuously deplete nutrients and change the environment composition (12, 13). However, in contrast to the soft-agar colony assays, localized microenvironments do not emerge during growth in continuously mixing liquid cultures (4).

The growth curves of wt *E. coli* and its isogenic mutant strains in the three tested nutrient conditions exhibit significant environment-dependent differences (*SI Appendix*, Fig. S1). For instance, a mutant strain behavior could be similar to that of the wt in nutrient-rich media, but dissimilar in nutrient-poor media (or vice versa). Another noticeable difference can be seen in the saturation value of the growth curve, which can be higher in growth media with low or medium nutrients than in the rich growth medium. This saturation value is determined by two factors: How efficiently the cell utilizes the nutrients provided in the medium, and given their efficiency, how many cells can be produced using the provided nutrients. Both elements are specific to the mutant and the medium it is growing in. Therefore, the saturation value can vary among the mutants and media used in a mutant-medium-specific manner and not necessarily always decreasing with the growth rate in the medium.

To quantitatively assess these differences, we extracted four parameters from the growth curves: maximum growth rate (MGR), saturation point of growth (SPG), area under curve (AUC), and time of exponential phase ($T_{\exp}$) (*SI Appendix*, Fig. S2*A*), all using established methods (11, 14). Some of the parameters, presented in *SI Appendix*, Fig. S2*B*, are strongly correlated for the wt and the 65 mutant strains in the three nutrient conditions (*SI Appendix*, Fig. S2 *C* and *D*) (see *SI Appendix*, *Supplementary Text 1* for a description of select details of the observed growth differences). Therefore, we performed principal component analysis (PCA) to obtain key attributes that identify mutants displaying similar growth profiles (*SI Appendix*, *Supplementary Text 4*). The resulting principal components indicate that approximately 95% of the variability among the mutant strains can be characterized using only two parameters (*SI Appendix*, Fig. S2*E*). The projection of the data points onto the first two principal components is shown in Fig. 1*A*, in which AUC-M9CG and AUC-M9G are the most relevant parameters to PC1, while AUC-LB, $T_{\exp}$-M9G, and SPG-LB are the most relevant parameters to PC2 (*SI Appendix*, Fig. S2*F*). Subsequent hierarchical clustering grouped the 65 mutant strains into two main clusters. Strains in cluster I (yellow circles in Fig. 1*A*) exhibit significant population growth differences from wt *E. coli* (black circle in cluster II). These consist mostly of enzymes of the cysteine metabolism pathway (*SI Appendix*, Fig. S3). The remaining single-gene deletion mutants in cluster II (orange circles in Fig. 1*A*) display less significant differences from wt *E. coli* in their growth profiles.

We note that single-subunit gene-deletion mutants of multisubunit protein complexes such as ATP synthase, or of metabolic pathway enzymes such as cysteine synthesis can exhibit significant variation in their growth dynamics. The structure of ATP synthase in *E. coli* consists of two main functional modules (Fig. 1*B*). The F_0_ module, composed of subunits *a*, *b*, and *c*, is embedded in the inner bacterial membrane, and is responsible for proton transfer. In contrast, the module F_1_, with subunits α‚ β, γ, ε, and δ, is membrane-extrinsic and is in the cytoplasm and carries out the proton gradient-driven ATP synthesis (15, 16). Although the roles of the ATP synthase subunits have been investigated in detail (17, 18), how bacteria adapt and compensate for deletion or inactivation of its individual subunits remains unclear. We find that the growth characteristics of ATP synthase subunit deletion strains are variable, though they all reside in cluster II (Fig. 1).

Such differences are not unique to multisubunit protein complexes like the ATP synthase. We observe more pronounced dichotomies among the growth of mutants with missing individual enzymes in metabolic pathways, such as the cysteine metabolism pathway (*SI Appendix*, Fig. S3). In this case, even when mutations affect the same multisubunit enzyme in that pathway, the effect of their deletions on *E. coli* can vary significantly. For example, *ΔcysU* and *ΔcysW* are two inner membrane subunits that are part of the sulfate/thiosulfate uptake system, yet *ΔcysW* exhibits more similar growth dynamics to the wt *E. coli* strain (cluster II in Fig. 1*A*) than the *ΔcysU* strain does (cluster I in Fig. 1*A*). These data imply that environmental conditions alter the growth of single-gene deficient bacteria in a complex, gene-specific manner.

### Adaptation to Deleterious Mutation-Induced Stress Is Associated with Global Reorganization of Gene Expression in an Environment-Dependent Manner.

When a random gene of *E. coli* is inactivated, *E. coli* cells routinely undergo complex, possibly evolutionarily conserved changes in their internal state to compensate for the loss of that gene product’s function and subsequent cellular stress, processes collectively referred to as allostasis (19–21). A major part of the allostatic response entails reorganization of the global gene expression profiles (transcriptome) of the mutated cells. These gene expression changes might reveal adaptive and/or homeostatic strategies that can account for similarities among the observed growth profiles. We therefore examined, in the three growth media, the transcriptome profiles of wt *E. coli* and a subset of the 65 single gene deletion strains, focusing on all eight subunit-encoding genes of the ATP synthase complex shown in Fig. 1*B* (*ΔatpA*, *ΔatpB*, *ΔatpC*, *ΔatpD*, *ΔatpE*, *ΔatpF*, *ΔatpG*, *ΔatpH*). We focus here on mutations in the same enzymatic complex in order to determine whether the transcriptome adaptation is determined by the function of the enzyme or is mutation-specific.

**Fig. 2.**
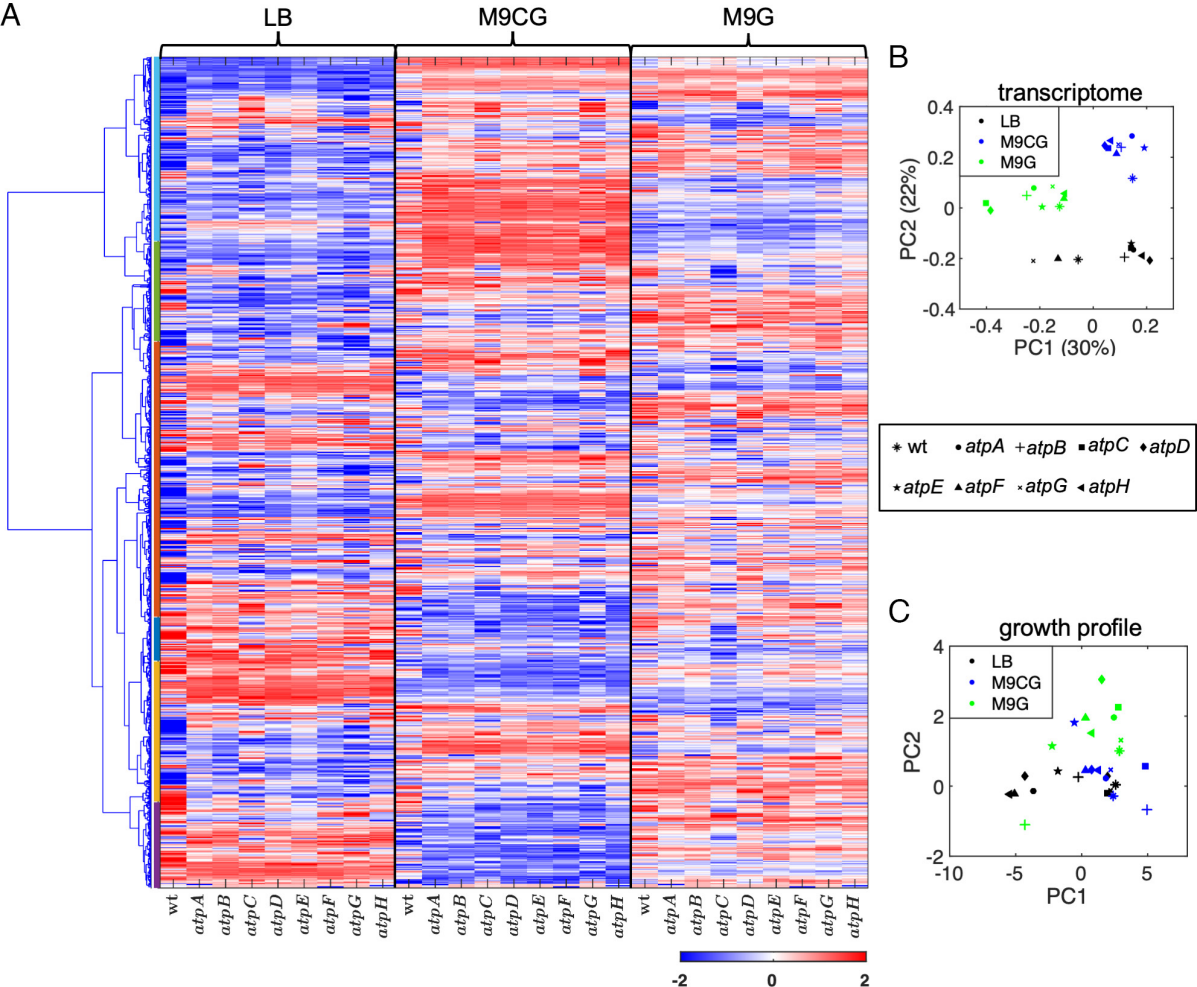
Global reorganization of gene expression in ATP synthase subunit deletion bearing strains. (*A*) Heatmaps of gene expression in all samples in three different media conditions at mid-log growth phase. Note: The similarities in gene expression profiles are pronounced within groups of samples from the same growth medium. The colors on the *Left* show clustering of the gene expressions into six clusters. See *SI Appendix*, *Supplementary Text 2* for detailed description of the obtained transcriptome profiles. (*B*) The expression profiles of all samples were subjected to PCA, and the resulting values of PC1 and PC2 are depicted. The samples are segregated into distinct clusters based on the media conditions. (*C*) The growth parameters (MGR, SPG, $T_{\exp}$, and AUC) were subjected to PCA in each media separately, and the values of PC1 and PC2 are shown here. Unlike the transcriptome PCA, overlap among the different media is observed.

Despite affecting the same protein complex (ATP synthase), these mutants displayed significant differences in their growth profiles (Fig. 1*B* and *SI Appendix*, Fig. S2*B*). The mid-log growth phase transcriptome profiles also displayed significant differences after average linkage hierarchical clustering by the variance of the top 1,021 features probed (Fig. 2*A*). This finding was further supported by the PCA across all the 10,208 features measured in all strains and in all three media (Fig. 2*B*). From these results, it is evident that the transcriptome profiles of these strains cluster mostly according to the environment (i.e., the type of growth media) in which they grow. Specifically, the transcriptome profiles of single gene deletion mutants growing in LB medium are more similar to the transcriptome profile of the wt *E. coli* growing in the same medium than they are to their own transcriptome profile when growing in different media (Fig. 2 *A* and *B* and *SI Appendix*, Fig. S4*A*). It is also evident that the clustering observed in the PCA of the transcriptome profiles (Fig. 2*B*) is significantly different from the clustering of growth profiles (Fig. 2*C*), which, unlike the transcriptome profiles, exhibit overlap between different media.

Nevertheless, granular analyses of the transcriptome profiles did uncover possible allostatic strategies that can account for the mutants’ ability to survive and function despite their gene losses. For example, our results reveal increased expression of genes used for anaerobic respiration such as *glpA*, *glpB*, and *glpC* in the LB medium, which together code for the anaerobic sn-glycerol 3-phosphate dehydrogenase (22). They also show an increase in the expression of genes responsible for the transport and/or production of different amino acids, e.g., the serine transporter, *sdaC*, and serine dehydratase, *sdaB*, in M9CG medium [these have been reported necessary for survival in glucose depleted environments (23)], or the prephenate dehydratase, *pheA*, in M9G medium (*SI Appendix*, Fig. S4*B*).

These results highlight the plasticity and resilience of bacterial regulatory networks that, in an environment-specific manner, allow for various adaptive strategies that activate alternate pathways to compensate for the loss of functionality due to inactivating mutations. Other changes in the transcriptome profile, including those with potential consequences for aging and senescence, are discussed in subsequent sections (*SI Appendix*, Fig. S4 and *Supplementary Text 2*).

### ATP Synthase α-Subunit Deficient *E. coli* Cells Increase Their Metabolic Activity.

**Fig. 3.**
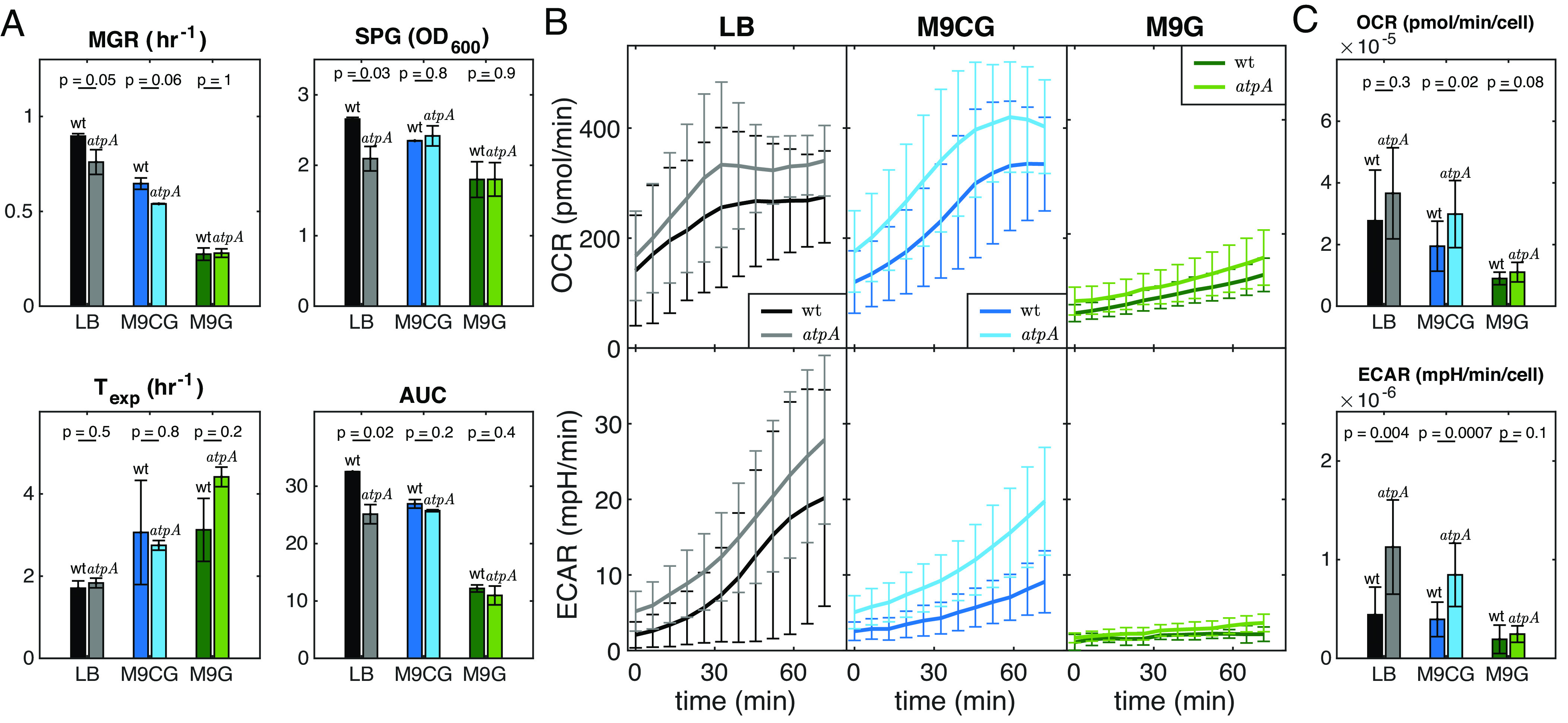
Enhanced metabolism in ΔatpA compared to wt *E. coli* cells. (*A*) The MGR, SPG, $T_{\exp}$, and AUC of wt and *ΔatpA* cells were compared in three different media, revealing a reduction in the differences between growth parameters of the two strains in low-nutrient environments. (*B*) OCR and ECAR of wt and mutant populations were compared in three different media over time. *ΔatpA* cells consume oxygen and glucose at a higher rate compared to wt *E. coli*, likely in order to compensate for their lower ATP synthase activity rate. (*C*) A comparison of calculated single-cell OCR and ECAR between wt and *ΔatpA* cells. The values in this panel were obtained by averaging and dividing the first three points of the graphs in (*B*) by the number of cells used in the experiments. All error bars are SDs. *P*-values in (*A*) and (*C*) were calculated using independent samples *t* tests.

To start clarifying how cells functionally adapt to gene inactivation-induced stress, we focused on the *ΔatpA* strain, which lacks the α-subunit of ATP synthase (Fig. 1*B*). This subunit is part of the catalytic head of the enzyme that phosphorylates ADP and is connected to the enzyme’s central stalk. Three active sites are formed at the interfaces of the three α- and β-subunits (24). It has been proposed that the β-subunit serves as the high affinity catalytic site responsible for binding ADP (25). Thus, *E. coli* cells may be able to synthesize ATP to some extent even in the absence of ATP synthase’s α-subunit. Indeed, the MGR of wt and *ΔatpA* cells are different in the three different media. In LB medium, wt and *ΔatpA* cells have significantly different growth rates; however, in media with lower nutrient availability (M9G), the growth rate of *ΔatpA* cells become more similar to that of wt cells (Figs. 1*B* and 3*A*, wt and *ΔatpA* have 15% difference in LB, 16% difference in M9CG, and 2% difference in M9G in their MGR). Similarly, the calculated SPG has comparable values for wt and *ΔatpA* cells only in environments where cells grow and proliferate at a slower rate (Fig. 3*A*, wt and *ΔatpA* have 21% difference in LB, 3% difference in M9CG, and 0.2% difference in M9G).

Our transcriptome analysis suggests that *ΔatpA* cells may upregulate both their respiration and glycolysis (*SI Appendix*, Fig. S4*B* and *Supplementary Text 2*). Therefore, we next investigated the relationship between cell growth, respiration, and glycolysis. To this end, we measured both the oxygen consumption rate (OCR) and extracellular acidification rate (ECAR) [proxies for cellular respiration and glycolysis, respectively (26)] in both *ΔatpA* and wt *E. coli* cells using the Seahorse technology (27) (*Materials and Methods*). It is evident that *ΔatpA* cells exhibit a higher OCR and ECAR than wt *E. coli* cells in the three growth media (Fig. 3 *B* and *C*). This difference was most striking in rich medium (LB) but was much less when grown in media with poorer nutrients (M9G) (Fig. 3 *B* and *C*). Indeed, the smaller differences in oxygen consumption and glycolysis rates between wt and *ΔatpA* strains are reflected in smaller differences between their growth dynamics as well (compare Fig. 3 *A* and *C*). This suggests that differences in growth dynamics between strains under different growth conditions are in part due to changes in their physiological state caused by the various mutations. In addition, these data show that mutant cells can sometimes compensate for suboptimal enzyme activity by increasing the activity of alternative pathways. For example, in M9G minimal medium, the *ΔatpA* strain appears to almost fully compensate for suboptimal ATP synthase activity, at least in part, by consuming oxygen and glucose at higher-than-normal rates. However, this compensatory, allostatic mechanism(s) is not sufficient to allow the *ΔatpA* strain to maintain identical population growth dynamics to that of the wt strain when grown in rich LB medium with fast cell growth.

### Growth and Division Dynamics of *E. coli* Cells Are Altered in Late-Stage Cell Generations.

Adaptation strategies employed by bacterial cells to counter specific environmental or intracellular (e.g., genetic mutation, epigenetic change, or protein damage) challenges can have various consequences for cell physiology. To better uncover how single gene deletion mutant strains adapt to loss of gene function, we next compared the single-cell growth and division dynamics of the representative *ΔatpA* mutant to that of the wt *E. coli* in the three growth media. For this, we utilized the experimental system known as the “mother machine,” which traps cells individually in a microfabricated array of channels (28) (see depiction in *SI Appendix*, Fig. S6*A*). In each experiment, *E. coli* cultures were started from a single agar-plate colony, grown in the desired medium to early exponential phase, and then loaded into the mother machine (*Materials and Methods*). This procedure ensured that all cells in each experiment were descendants of a single mother and were closely related at the time of the measurement. Trapped cells carrying plasmids expressing fluorescent proteins were tracked as they grew and divided until they stopped proliferating. While the addition of plasmids does affect the cells’ growth characteristics, this effect is similar for all mutants and growth conditions, and therefore, all the parameters of the population growth curves maintain strong linear correlations (*SI Appendix*, Fig. S5). This allows for reliable comparison between the growth dynamics of single cells containing the plasmids with that of the population without the plasmid, as we discuss below.

**Fig. 4.**
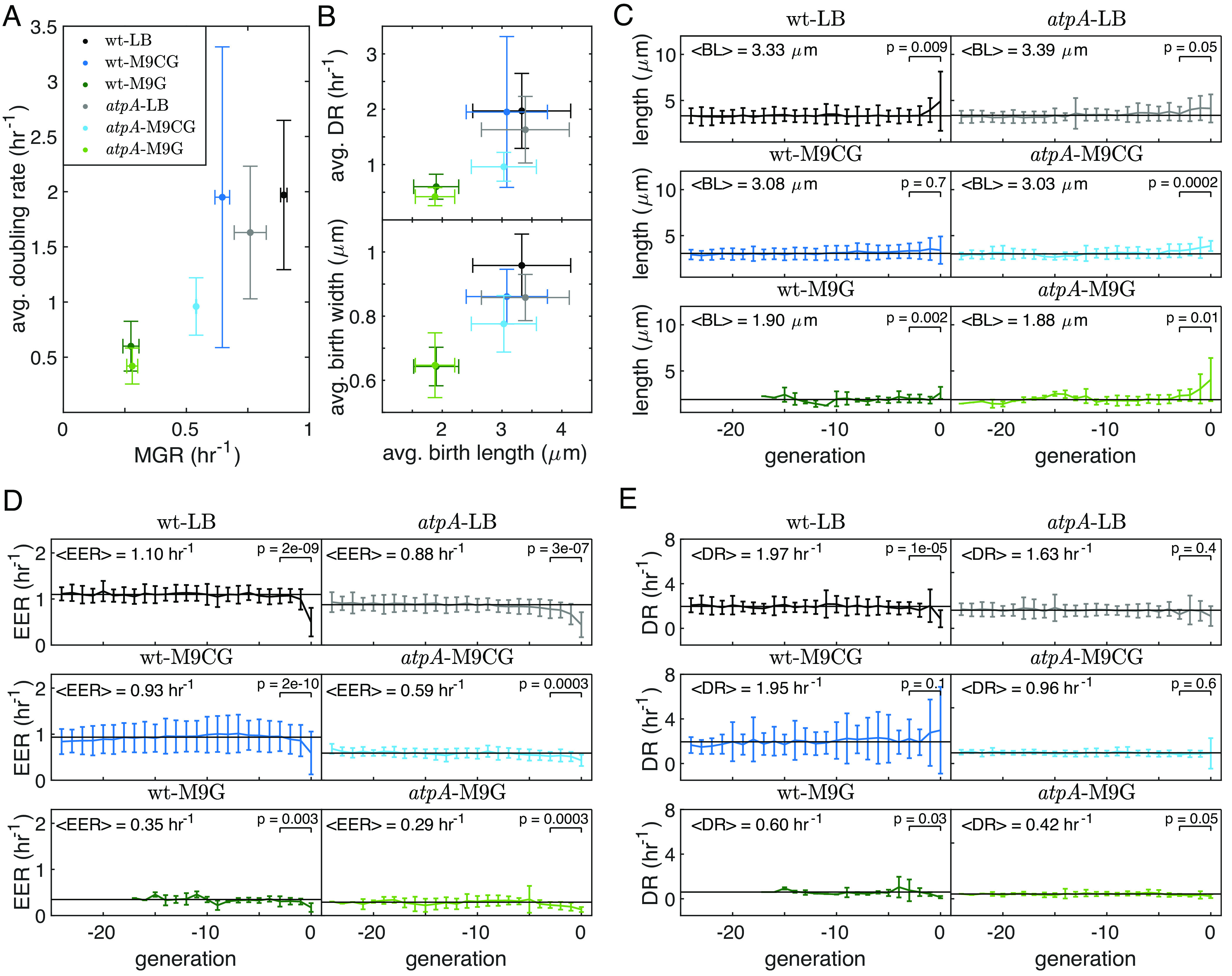
Alterations in the final cycles of replicative lifetime revealed by single-cell growth dynamics. (*A*) The average DR of single cells, which is the inverse of the cell cycle duration, is positively correlated with the MGR of the population. (*B*) The average DR of single cells (*Top* graph) and the average birth width (*Bottom* graph) are positively correlated with the average BL in different media. (*C*) BL, (*D*) EER, and (*E*) DR of single cells during the last ∼25 generations of their replicative lifetime in different nutrient environments. A deviation from the average BL and EER is observed in the last generations. The straight horizontal black lines in each of the plots represent the average of the entire population over all generations except the last three, and their values are depicted in each of the plots (<.> denotes the average value). Error bars represent SDs. *P*-values were calculated using the paired *t* test between generations 0 and −4. The number of cells analyzed in each condition were $N_{wt-LB}=32$, $N_{wt-M9CG}=75$, $N_{wt-M9G}=16$, $N_{atpA-LB}=32$, $N_{atpA-M9CG}=40$, and $N_{atpA-M9G}=16$.

Proliferation of cells was characterized by their time-dependent length variation, from which their exponential elongation rate (EER) and doubling rate (DR) were calculated (see single-cell data analysis in *SI Appendix*, Fig. S6*B* and *Supplementary Text 4*). As expected, a comparison of the average DR of the wt and *ΔatpA* cells in the different growth conditions with their population MGR under the same conditions shows a strong correlation (Fig. 4*A*). This indicates that the population growth dynamics reflects the average single-cell proliferation rate. Additionally, it has been reported that *E. coli* cell size and growth rate decrease in nutrient-poor conditions (29). Our measurements also show that the average birth length (BL), average DR, and average birth width of cells are similar for wt and mutant cells and decrease in M9G media and are correlated with each other (Fig. 4*B*). These results demonstrate that *ΔatpA* cells preserve the same homeostatic mechanism(s) as wt *E. coli* cells, but with a slower proliferation rate in nutrient-limited medium.

We also examined the distributions of the DRs in the different conditions (*SI Appendix*, Fig. S6*C*). The results show that the coefficient of variation ($\text{CV}=\frac{\mathrm{SD}}{\text{mean}}$) for the wt strain increases with the stress level due to the decrease in the quality of the growth medium. On the other hand, the CV for the mutant does not change notably. In previous studies, it has been shown that stress can increase growth rate variation and even induce growth arrest, especially in cases of metabolic imbalance and/or accumulation of some metabolites in the cell (30, 31). Interestingly, we do observe that most wt cells exhibit a decrease in their DR when grown in the medium- and low-nutrient growth media, but some still exhibit a high DR as in rich growth medium. In the case of the mutant cells, the decrease in the DR appears to be global since the CV also remains the same in the medium- and low-nutrient growth media as in the rich growth medium. This suggests that the mutation might be affecting the cells’ ability to adapt to nutrient depletion. How and why this happens requires deeper investigation into the variation of the transcriptome profiles among cells.

In addition, we tracked the change in growth rate and cell size along the cell’s lifespan. Previous studies have consistently shown that the proliferation rate of *E. coli* is stable over hundreds of generations (28, 32). To verify this, we extracted the cells’ BL, EER, and DR in the last ∼25 replication cycles in all three media. Because cells enter the traps at different ages, their actual age is unknown to us. To better identify possible aging-related effects, we aligned our data relative to the last replicative cell cycle. These results are presented in Fig. 4 *C*–*E*, respectively, where generation zero is the last cell cycle with a nonzero growth rate. It is evident that the BL, EER, and DR are maintained even late in the cells’ replicative life and change only during the final stages of replication. Our results reveal a potential signature of aging-related stress response toward the end of the cell’s replicative lifespan, which appears as an increase in the cell’s BL (Fig. 4*C*) and a decrease in its EER (Fig. 4*D*).

### Replicative Crisis Drives *E. coli* Cells into a Postreplicative State Prior to Cell Death.

Another key finding of our single-cell analyses was that wt and *ΔatpA* mutant *E. coli* cells did not disintegrate and die once they stopped proliferating. To quantify the exact time of death relative to the cell’s proliferation and DNA replication arrests, we introduced the GFP-fused DNA-binding protein (GFP-Fis) in the cells and added the red dye propidium iodide (PI) to the medium circulated through the mother machine. The GFP-Fis, which is constitutively expressed in cells, binds to DNA, thus allowing us to track DNA replication in the cell. The PI, on the other hand, can enter the cell when the plasma membrane becomes permeable indicating imminent cell death (33). Consequently, fluorescence images of GFP-Fis and PI in cells as they grow and divide provide a much clearer picture of single-cell physiology and aging dynamics. Multiple experiments were performed for each growth condition. The detailed results are presented in *SI Appendix*, Table S2.

**Fig. 5.**
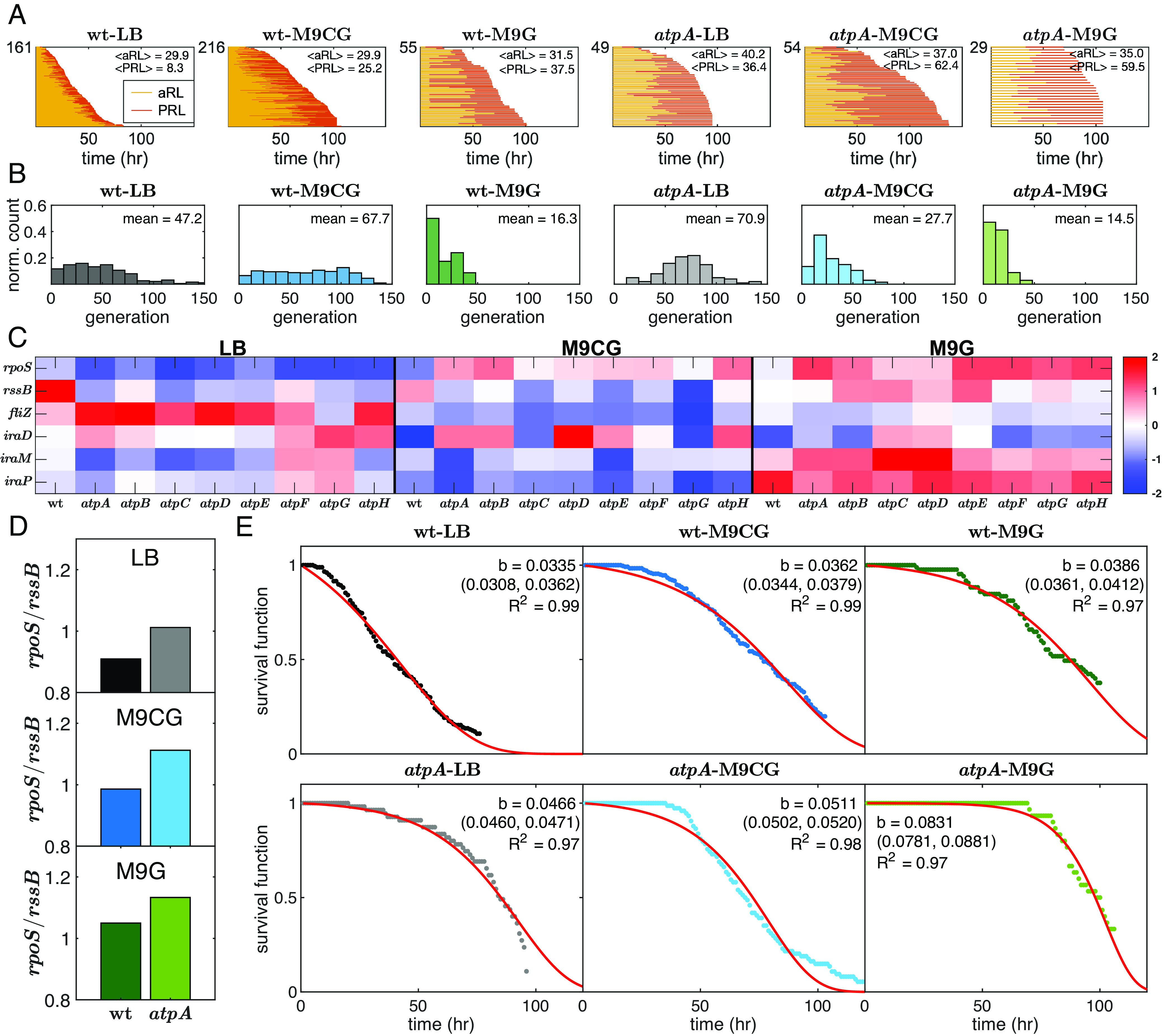
The effects of mutation-induced stress on bacterial lifespan and aging. (*A*) Distributions of the aRLs and the PRLs of wt and *ΔatpA* cells in three different growth media are shown. Each line represents one cell, and the total number of cells is indicated on the *y*-axis. Growth in poor media leads to an increase in PRL. <.> denotes the mean. (*B*) Distributions of aRL in terms of the number of generations. Cells undergo fewer generations in stress conditions. (*C*) Heatmap of gene expressions of *rpoS*, *rssB*, and other genes related to them. Nutrient limitation in M9CG and M9G leads to an increase in *rpoS* expression and a decrease in *rssB* expression. (*D*) The ratio of *rpoS* to *rssB* expression increases under stress conditions. (*E*) Survival functions of wt and *ΔatpA* in three media conditions. Dotted lines represent the experimental data, while the red lines are a fit to a Gompertz equation, allowing the estimation of aging rate (b) as shown in each graph. The aging rates are presented in units of h^−1^ and the numbers in the parentheses present 95% confidence bounds. Under stress conditions, there is an increase in aging rate.

The use of PI revealed that following the last cell division, *E. coli* cells entered a nonreplicating state reminiscent of replicative senescence in mammalian cells. We call this segment of the cell’s lifespan (from the end of proliferation until PI entry) its “postreplicative” lifespan (PRL). Likewise, we refer to the proliferation period as the cell’s “apparent replicative” lifespan (aRL). This is because the actual replicative lifespan of a cell is not known (as we discussed earlier, the number of replications since the birth of a trapped cell at the beginning of the experiment is unknowable in this experimental setup). However, due to the high number of data points in each experiment, the distribution of these values should represent the entire population well. Our results show that the PRL is prolonged in the *ΔatpA* mutant relative to the wt strain (Fig. 5*A*), and that entry into PRL is accelerated, in terms of number of cell generations, in nutrient-limited media (Fig. 5*B*). This prolonged PRL is intriguing. An initial clue why it may happen comes from our microarray analyses (*SI Appendix*, *Supplementary Text 2*), which reveal a general stress response in the *ΔatpA* mutant strain and in nutrient poor media. This response is controlled by the master transcriptional regulator, RpoS, which is known to be activated by nutrient deprivation or molecular damage (34). Previous research has demonstrated that decreasing the availability of RpoS in the cell by deleting its coding gene, *rpoS*, increases the cellular aging rate in nonreplicating cells under deprived nutrient conditions (35–37). Conversely, increasing its availability by deleting the *rssB* gene, coding for the stationary-phase regulator, RssB [which is essential for the RpoS degradation (38, 39)], extends the lifespan of nonreplicating cells (35). The expression profiles of the two strains in the tested media show that the prolonged PRL coincides with either increased *rpoS* expression, or a decreased *rssB* expression (Fig. 5*C*), or both (Fig. 5*D*). Additionally, we observed both a reduction in the gene expression of *fliZ*, an antagonist of *rpoS* that is downregulated during the cellular stress response (40), and an increase in the gene expression of *iraP*, *iraM*, and *iraD*, the products of which are RpoS stabilizers during Pi starvation, Mg starvation, and after DNA damage, respectively (41) (Fig. 5*C*). Note that for the general stress response to be activated, not all these changes need to occur simultaneously, but rather any subset of these changes that would result in more active RpoS, would lead to the activation of the stress response. Therefore, the transcriptome profiles of these genes vary among the different strains and conditions, but the direction of change is such that there are relatively more active RpoS proteins, as demonstrated by the ratio of *rssB/rpoS* in Fig. 5*D*. A more complete understanding of this phenomenon, however, requires further identification of its underlying molecular control mechanisms.

### Environmental and Genetic Stressors Accelerate Bacterial Aging.

The observed increase in BL (Fig. 4*C*) and decrease in proliferation rate (Fig. 4*E*) toward the end of the replicative lifespan, as well as the accelerated entry into the PRL phase (Fig. 5*B*) suggest that further examination of the effects of adaptation strategies on the aging of *E. coli* cells is needed. Therefore, we next compared the survival distributions of both *ΔatpA* and wt *E. coli* cells in the three different growth media (Fig. 5*E*). These distributions describe the Kaplan–Meier survival probability function estimated by $S(t)=\prod_{t_{i}\leq t}[1-d_{i}]$, where t is time and $d_{i}$ is the fraction of cells that die at time $t_{i}$. The survival function, as has been shown in previous studies, is best described by the Gompertz distribution: $S\left(t\right)=e^{-\frac{a}{b}\left(e^{\mathrm{bt}}-1\right)}$, where S is the survival function, t is time, and a and b are constants. From the survival function, the hazard function (or mortality risk) can be calculated as $\lambda \left(t\right)=-\frac{\dot{S}\left(t\right)}{S(t)}=ae^{\mathrm{bt}}$. The increase in $\lambda \left(t\right)$ with time signifies aging, with *b* depicting the aging rate (or hazard accumulation rate), which is less than 1, since a rate of 1 or higher would lead to the extinction of the population (42).

Although molecular mechanisms of aging in bacteria have yet to be identified, previous studies have demonstrated an increase in the hazard function of wt *E. coli* cells over time (28, 35, 43). In agreement with these studies, we observed aging in all our experimental conditions, irrespective of the medium or the strain used. The values of aging rates (*b*) obtained from our measurements are presented in Fig. 5*E* (for the values of all experiments separately, see *SI Appendix*, Table S2). These values demonstrate that the *ΔatpA* mutant strain’s aging rates are generally higher than those of the wt strain and increase for both strains as the growth medium becomes poorer with nutrients.

We also examined the survival functions by considering the total number of generations within the duration of the cells’ aRL, rather than their total lifetime, revealing significant differences (*SI Appendix*, Fig. S6*D*). The aging rates per generation during the aRL of both wt and *ΔatpA* cells in the rich and extremely poor medium exhibit roughly similar ratios as those observed in the aging rates obtained from the whole lifespan.

The survival function analysis and the aRL and PRL measurements presented above determine the probabilities of bacterial division, growth arrest, and death. These have consequences for the population growth dynamics, which we discuss and model in *SI Appendix*, Fig. S7 and *Supplementary Text 3*.

### Single Cells Display Diverse PRL Morphology Phenotypes.

**Fig. 6.**
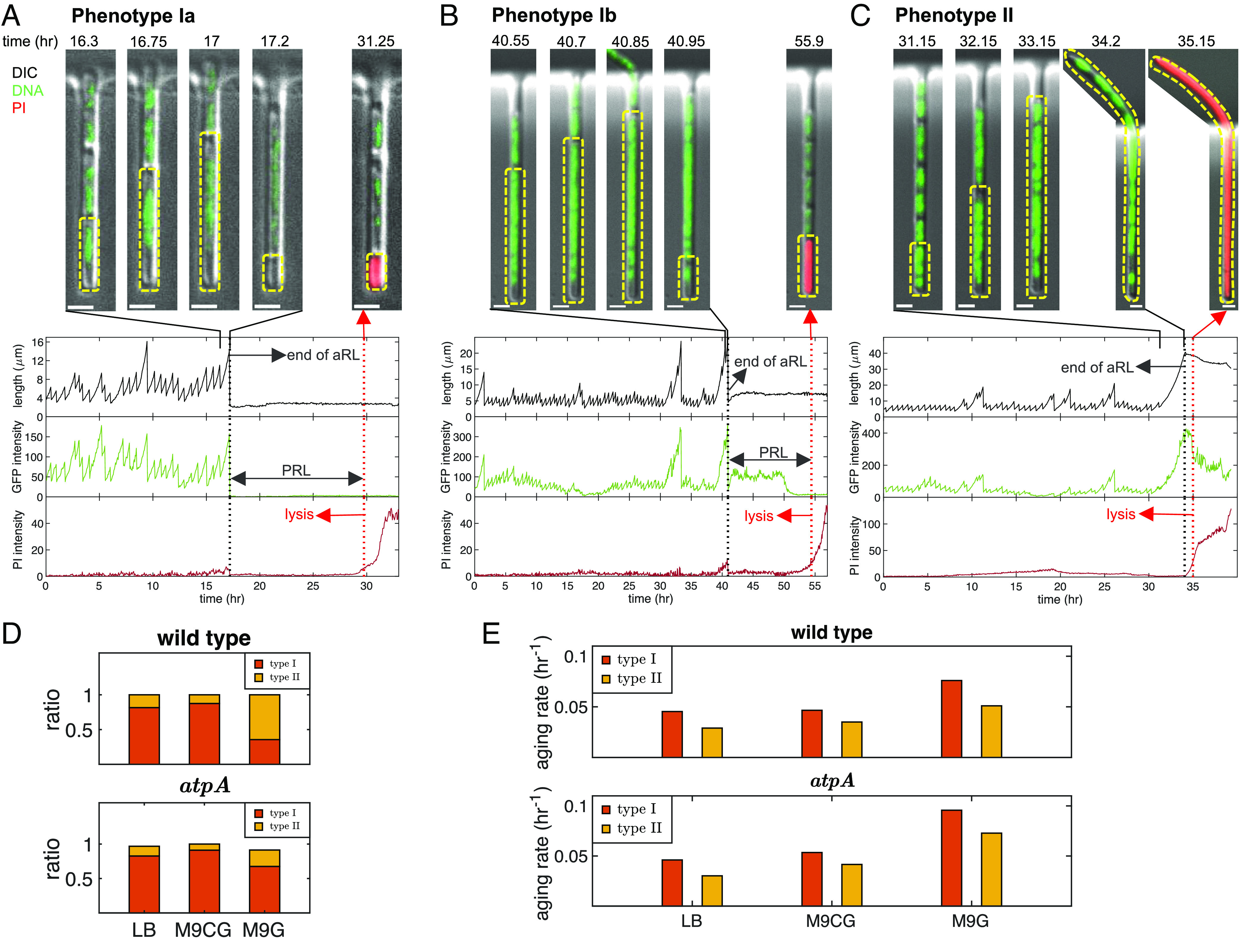
Single-cell experiments reveal different morphological PRL phenotypes and aging rates. (*A*) Example of phenotype Ia, showing images of the last replicative generation and the beginning of lysis. The cell length is plotted over time, showing its aRL and PRL. The GFP intensity reveals the absence of DNA after the last division, but the cell remains alive during the early stages of PRL before PI dye entry, revealing lysis. (*B*) Example of phenotype Ib, showing a gradual decrease in GFP intensity during the PRL before PI dye entry and lysis, indicating the presence of DNA during the last division. (*C*) Example of phenotype II, showing an elongated (filamented) cell that remains in this state until lysis. Scale bar are 2 µm. Yellow dotted lines on the images are the outline of the mother cell. Black dotted lines on the graphs indicate the end of aRL, while the red dotted lines indicate lysis. All three examples are wt cells grown in LB. (*D*) Percentages of different phenotypes in wt and *ΔatpA* under three media conditions. The percentage of phenotype II cells is increased under stressed conditions, revealing a phenotypic change strategy due to stress. (*E*) The aging rates of different phenotypes in wt and *ΔatpA* under three media conditions calculated in *SI Appendix*, Fig. S8. Phenotype II cells exhibit lower aging rates compared to phenotype I cells revealing a stress effect on switching phenotypes.

The images of cells in the mother machine reveal that in addition to exhibiting variable growth rates and cell sizes, cells also exhibit different modes of senescence and death. Specifically, we identified two different PRL phenotypes based on the cells’ morphology and behavior toward the end of their lives. Fig. 6 *A*–*C* present examples of these phenotypes (Movie S1). In cells with phenotype I, cell size after the last division is similar to the average population size. By additionally tracking the chromosome in each cell through GFP-Fis fluorescence, we were able to divide phenotype I into two subcategories (excluding cells that lose their plasmid, see *SI Appendix*, *Supplementary Text 4*, last column of *SI Appendix*, Table S2 and Movie S2). Phenotype Ia cells lose their chromosome during the last division (illustrated by the sudden loss in the GFP intensity at 17.2 h in Fig. 6*A*). This event drives the cell into the PR regime, where it cannot grow but can still perform some activities that do not require the chromosome (44). Phenotype Ib cells preserve their chromosome throughout their entire lifespan, but still enter a nondividing, postreplicative phase before lysis (Fig. 6*B*). In both cases, the increase in the PI intensity in the cell marks the onset of cell lysis and death. In contrast, phenotype II cells enter a replicative crisis during their last generation, characterized by the cell’s excess elongation and slower growth rate (Fig. 6*C*) - a well-known phenotypic stress response in bacteria called filamentation (45) (we consider any cell whose length exceeds 3x the average cell length in the tested medium to be filamented). In this subpopulation, DNA replication continues normally during the last cycle, but it occurs in the absence of cell division. Filamentation occurs often during any cell’s aRL. This usually triggers the cell’s stress response, which corrects for the cause of filamentation (46). Here, however, cells fail to respond properly, which in turn drives the cell into its PRL.

The aRL and PRL distributions of each strain when classified into these two phenotypes are presented in *SI Appendix*, Fig. S8, where entire lifespans are presented as a function of time (*SI Appendix*, Fig. S8*A*) or the aRL is presented as a function of number of generations (*SI Appendix*, Fig. S8*B*). The fraction of each phenotype in the two strains growing in different growth media is shown in Fig. 6*D* (for more details see *SI Appendix*, Table S2). It is evident that a higher proportion of cells exhibit phenotype II morphology in the nutrient-limited M9G medium than in media with rich (LB) or average (M9CG) nutrient content and that phenotype II is more common in wt than in *ΔatpA* cells. This suggests that phenotype II reflects the adaptation of cells to nutrient-poor environments, which can induce a general stress response in cells (47). Indeed, the transcriptome profiles show that in M9G medium, where phenotype II is prevalent, expression of the general stress response regulator *rpoS* increases by 2.5x in the *ΔatpA* strain compared to its expression in LB medium. In contrast, in the wt strain, the expression of *rssB* required for RpoS proteolysis decreases by 2.3x in M9G medium compared to the LB medium (Fig. 5*C*). In both cases, the end result is increased cellular levels of RpoS, which induces a general stress response. For more details on how the stress response leads to a different cellular morphology prior to death, see *SI Appendix*, *Supplementary Text 2*.

We next investigated aging rates of cells in the two PRL phenotypes. Fig. 6*E* presents the aging rates of each phenotype in the three different growth media for both wt and *ΔatpA* cells, extracted from the survival functions illustrated in *SI Appendix*, Fig. S8*C*. In all cases, it is evident that phenotype II cells have lower aging rates compared to phenotype I cells. This result suggests that entering into a replicative crisis and undergoing filamentation, as exhibited by phenotype II cells, may be a way to protect cells from aging. This provides further evidence that most phenotype II wt cells undergo a phenotypic switch induced, in turn, by the allostatic response that helps cells adapt to environmental stress. Further studies are needed to clarify the molecular mechanisms underlying the observed differences in aging rates between the two death phenotypes.

## Discussion

In microorganisms, ongoing random mutagenesis enables their adaption to new environments by creating a genetically diverse cell population upon which natural selection can act (48). Similarly, in humans, genomic heterogeneity is widespread from embryogenesis to old age both in normal and diseased tissues (49–51), including loss-of-function mutations (52), at least in part to allow for functional selection during embryogenesis and to counter tumorigenesis and aging-related tissue dysfunction (53–57). However, the potential functional cost of maintaining such evolutionarily conserved cellular capabilities remains poorly understood.

In this study, we have investigated how *E. coli* cells survive and adapt to the presence of metabolic gene-inactivating mutations by characterizing the population growth of multiple single-gene deletion *E. coli* strains, as well as their growth and proliferation at the single-cell level, in different environments. We have found that while some genes’ inactivation has almost no consequence for the population-level proliferation, other single-gene deletions attenuate it in an environment-dependent manner. For example, we observe dissimilar population growth of the ATP synthase α-subunit deletion mutant (*ΔatpA*) and wt *E. coli* in a nutrient-rich medium, while they display similar growth profiles in nutrient-poor media. One possible reason for this discrepancy is that cells carry out multiple processes in tandem in order to grow and proliferate. For example, in a nutrient-poor medium, the cells are expected to produce metabolic products unavailable for uptake from the medium yet required for cell growth. This in turn can significantly reduce the growth rate of the cell, and thus suboptimal ATP synthase activity could become sufficient to provide the needed ATP for slow bacterial growth. In contrast, in a rich medium many of the products required for the cell’s growth are already present in the medium, allowing faster growth. However, in such growth environments, suboptimal ATP synthase activity may become a limiting factor for the cell’s rapid growth, even when complemented by other ATP-producing processes like glycolysis.

In addition, the obtained gene expression profiles reveal global changes that are gene-deletion and/or environment specific. These changes allow cells to better survive the challenges they face. This widespread gene expression reorganization in all tested mutant strains suggests that conserved homeostatic buffering mechanisms (i.e., allostatic programs) have been activated to compensate for the detrimental effect of gene deletions. Part of this reorganization includes the expression of gene products that compensate for the loss of functionality due to the mutation. An example of this is the case of *ΔatpA* mutants, where the reduced efficiency of ATP synthase, resulting from the deletion of this enzyme complex’s α-subunit, is marked by a significant increase in the respiration and acidification rates of the mutant cells. In this case, the reduced efficiency of the ATP synthase is in part compensated by the expression of genes used in anaerobic respiration that we further confirmed by functional metabolic studies (Fig. 3). In other cases, the reorganization of gene expression profiles includes the expression of the general stress response in the cell, which is normally expressed under starvation or molecular damage conditions.

These results indicate that their regulatory network plasticity, potentially involving the altered expression of both canonical and noncanonical cellular components -from moonlighting proteins to transient translatomes (58–62), allows cells to adapt to various environments and even compensate for the loss of some gene functions (63). In turn, this adaptation allows nonlethal deleterious mutations to persist within the population for an extended time. This adaptation, however, is not without cost to the health of the cell. Our examination of two strains, namely the wt and the *ΔatpA*, in three media with different levels of nutrients abundance, reveals that *E. coli* cells growing under more challenging conditions, whether the challenge is due to reduced nutrients or severe reduction or loss of a gene’s function, experience accelerated aging in the replicative regime of their lifespan and reduced functionality during their postreplicative lifespan.

These findings show remarkable similarities to that seen in select neurodegenerative and genetic diseases in humans. For example, humans with Huntington’s disease carry expanded CAG repeats in their Huntingtin (*HTT*) gene and aberrant Htt function from conception but only start to exhibit clinical symptoms at early to mid-adulthood. This suggests that their affected cells are able to buffer the deleterious effect of the abnormal htt for decades (64). Similarly, the cells of human patients with some inherited primary mitochondrial oxidative phosphorylation defects display high rates of mitochondrial respiration, greatly increased rate of glycolysis, and reduced lifespan (65, 66), phenotypes quite similar to that observed here in *E. coli* cells lacking their ATP synthase α-subunit (Fig. 3). Also, the role of acquired mitochondrial dysfunction and stress in the pathogenesis of select neurodegenerative diseases such as Parkinson’s disease is increasingly recognized (67, 68). These parallel findings imply that some bacterial adaptability mechanisms may have remained conserved in eukaryotes, including in humans, at times manifesting themselves as contributing factors to genetic or somatic disease and reduced lifespan (52, 66, 69).

**Fig. 7.**
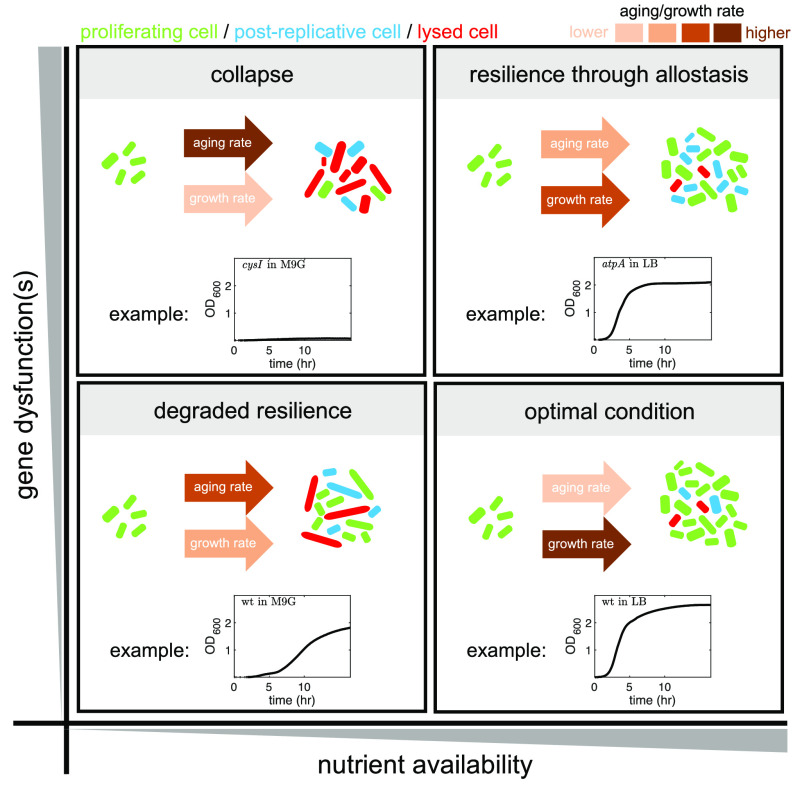
Model for functional resilience in cells with deleterious gene mutations. Genomic heterogeneity allows long-term adaptation of bacterial cells to new environments but at a cost of many cells carrying deleterious mutations. Stress-induced homeostatic (allostatic) mechanisms may be able to fully or partially compensate for gene inactivation-mediated loss of canonical protein functions, but at a cost of increased filamentation, accelerated aging, and decelerated growth, manifested as extended postreplicative state and/or earlier lysis and death. The level of achievable allostasis is environment dependent. The rate of cell proliferation and aging and their relative ratio determine the apparent cell growth rate at the cell culture level. Proliferating cells (green), cells in postreplicative lifespan (blue), and lysed cells (red) are shown.

The meaning and mechanisms of aging in single-cell organisms, such as bacteria, have been debated for several decades. Initially, symmetrically dividing unicellular bacteria were thought to be immortal, and their death was thought to occur due to factors other than aging, such as injury or disease (70). Recent studies, however, have identified aging markers in such bacteria, by recognizing that one of their cell poles is created anew during every cell division, while the other pole remains the same and therefore ages with every division event (71). This insight has since been used to measure bacterial age by tracking the effect of pole aging on physiological factors such as the inheritance of protein aggregates (72), growth rate (71, 73, 74), and survival probability (35, 75, 76). While the growth rate has not shown any trend depending on cell age (28), comparing the doubling time of sister cells reveals that the sister that inherits the maternal “old” pole exhibits longer doubling time (77). Additionally, inheritance of protein aggregates has been found to colocalize with the aging pole (72). Here, we uncover additional bacterial aging characteristics and the influence of loss of gene function on them. Our examination of a representative deleterious mutation (*ΔatpA*) at the single-cell level reveals that individual mutant cells are able to maintain near-normal growth and division, yet at a lower average growth rate. Before lysis, they enter a postreplicative state of their lifespan, where though their growth is arrested, they remain alive. The probability of a cell entering this phase increases with its age and varies depending on the nutrients available in its environment. Their survival in this postreplicative regime appears to be assisted by the expression of the general stress response. This can be inferred from the cells’ transcriptome profiles, which show either an increase in the expression level of the stress response regulator *rpoS* or a decrease in the expression of *rssB* required for RpoS degradation (or both). In either case, the result is an intracellular increase in the RpoS, which activates a stress response. We also find that the activation of the stress response is accompanied by accelerated aging and a change in the death phenotype. These events are observed both in the presence of a deleterious mutation, such as *ΔatpA*, as well as when nutrients become scarce in the growth medium. We have also found that cells tend to filament during their last cell cycle before entering the PRL when the stress response is triggered. Although filamentation has been previously reported as a stress response, the details of how and when such event occurs remain vague (27). The inhibition of cell division has also been observed as a result of environmental stresses, such as pressure (78), antibiotic exposure (79), or interaction with hosts (80). The relative dominance of the stress factors determines the fate of bacteria, which ranges from optimal growth to growth collapse and cell death (Fig. 7). In addition to the stress response, intended to alleviate stress effects and restore cellular health, stress can also have ramifications as proliferating damage affecting various physiological functions. This in turn can lead to cell death (43) and might be the factor driving the accelerated aging we report here. Further research is needed to uncover the generality of the effects on aging detected here, as well as to disambiguate and disentangle the two effects (ramifications and allostasis) from one another and establish a direct molecular connection between the stress response (or stress ramifications), aging, and death phenotypes in bacteria. Such studies can also shed light on how such insights can be utilized for clinical purposes. For example, the single or combined pharmacological inhibition of gene products whose deletion leads to accelerated aging in bacteria may represent potential bacteriostatic antibiotic targets. Similarly, compounds affecting allostatic programs in select human diseases may also be able to be developed.

## Materials and Methods

### Bacterial Strains, Growth Conditions, and Reagents.

*E. coli* K-12 BW25113 and its isogenic mutant derivatives were obtained from the Keio *E. coli* mutant collection (81). A complete list of all used strains is presented in *SI Appendix*, Table S1. The cells used in the mother machine experiments carried two additional plasmids: pZA3R-mcherry (44) the chloramphenicol-resistant medium copy-number plasmid pZA under the control of the constitutive viral $\lambda -P_{R}$ promoter, and PRJ2001-GFP-Fis which was used for chromosome labeling [a kind gift from the Marko Lab, Dept. of Molecular Biosciences, Northwestern University, Evanston, IL 60208-3500 (82)]. Three different growth media were used in the experiments: Luria Broth (LB, used as a rich nutrient condition for rapid growth), M9 minimal medium supplemented with 1 g/L casamino acids and 4 g/L glucose (M9CG, a moderate nutrient condition used for average growth), and M9 minimal medium supplemented with 4 g/L glucose (M9G, a nutrient-poor condition used for slow growth). Depending on the strain being used, either 25 µg/mL kanamycin, or 30 µg/mL chloramphenicol, or both, were added to the medium.

### Microbatch Culture Experiments.

All isogenic single gene deletion mutants and wt *E. coli* strains were stored in microtiter plates at −80 °C. On the day before the experiment, strains were picked from frozen stocks and grown overnight at 37 °C with agitation at 240 rpm in the selected medium. The overnight cultures were then further diluted to optical density (OD_600_) ∼ 0.001 in fresh medium. 250 µL of each cell type was then transferred into a 96-well cell culture plate in triplicates and grown up to 16 h at 37 °C in a plate reader (Infinite 200, Tecan Trading AG, Switzerland). The growth of the various cell-type populations in the different growth conditions was characterized by obtaining optical density measurements at 600 nm every 5 min with shaking in between measurements. All experiments were repeated three times.

### Microarray Sample Collection.

Cells were grown from frozen stock overnight in LB, M9CG, and M9G. The next morning, they were diluted and grown to exponential phase (OD = 0.3 in LB; OD = 0.2 in M9CG; OD = 0.1 in M9G), and 0.5 mL of the LB and M9CG samples, and 1 mL of the M9G sample were collected. All samples were mixed with 2× volume RNAprotect Bacteria Reagent (QIAGEN) and centrifuged at 10,000 rpm for 10 min. The supernatant was decanted, and the pellet was stored at −80 °C. The next morning the pellet was lysed by adding 100 µL TE buffer (1 mL of 1 M Tris-Cl, 200 µL of 0.5 M EDTA, 98.8 mL dH_2_O) with 1 mg/mL lysozyme. The RNA was extracted using the RNeasy Mini Kit (QIAGEN). Samples were sent to The Boston University Microarray & Sequencing Resource for Genome array and 3'IVT Pico processing and Bioanalyzer QC (www.gg.bu.edu/microarray/index.htm).

### OCR and ECAR Measurements.

OCR and ECAR were measured using a Seahorse assay modified for bacterial cells (27). The wells of a 96-tissue well microplate (Agilent) were coated by poly-L-lysine [0.1% (w/v) Poly-L-lysine from Sigma-Aldrich] in order to bind and immobilize the bacteria in preparation for the measurements. This was achieved by adding 15 µL of 0.0004% Poly-L-lysine and leaving the plate in a hood overnight to allow the evaporation of the solution. The following day, each well was rinsed with dH_2_O to remove excess poly-L-lysine molecules. This procedure has been shown to have a negligible effect on the metabolism of *E. coli* (83).

Cells were grown in LB overnight in an incubator at 37 °C with shaking at 240 rpm. The following day, they were diluted 100× in the three different media and regrown until they reached exponential phase. The cultures were then diluted further to OD_600_ ∼ 0.02 in fresh medium, 90 µL of each culture was added to the poly-L-lysine coated wells of the microplate in triplicate wells, and the plate was centrifuged for 10 min at 4,000 rpm. An additional 90 µL of fresh medium was then added to each well. In addition, three wells were filled with clear media (no cells) only for control, and the plate was incubated for 1 h before placing it in an Agilent Seahorse XFe96 Analyzer for measurements. Cells’ concentration at the beginning of the measurement was estimated from bright field images of the wells and using imageJ (84) for cell counting. This concentration was used to normalize the measurements in Fig. 3*C*.

### Single-Cell Experiments in Mother Machine.

Cell length and protein expression were measured as previously described (32), using the “mother machine” (28). Two different strain formats were used: one containing only the PZA3R-mcherry plasmid and the other containing both the pZA3R-mcherry and PRJ2001-GFP-Fis plasmids. The wt and Δ*atpA* strains with the plasmid(s) were grown from an agar plate overnight at 37 °C with agitation at 240 rpm in the selected medium with appropriate antibiotics. The overnight cultures were diluted 100× in fresh medium and grown at 32 °C until early exponential phase, OD_600_ *~* 0.1 to 0.2. Cells were then concentrated into fresh medium to an OD_600_ ~0.3 and loaded into the trapping device shown in *SI Appendix*, Fig. S6*A*. The microchannels had a width of ~1 µm, which is larger than the widths of the strains grown in all media that were used in this study, ensuring that the differences observed in the growth rates are not due to lack of nutrients. The device was either mounted on a Zeiss Axio Observer microscope or on a Nikon eclipse Ti2 microscope with a 100× objective. Temperature was maintained at ~30 °C using an in-house-made incubator for the Zeiss microscope, and a microscope top incubator (Okolab, H201-1-T-UNIT-BL) for the Nikon microscope. Fresh medium was pumped through the device at a rate of 1 mL/h throughout the experiment. All media contained 1 mM Isopropyl β-D-1-thiogalactopyranoside for GFP-Fis induction. Propidium iodide (IP) from LIVE/DEAD^TM^ *Bac*Light^TM^ Bacterial Viability Kit (Thermo Fisher) was added to the medium to identify dead cells. When IP was used, the plasmid pZA3R-mcherry was not, to avoid fluorescence cross-reading. Images of the channels were acquired every 3 to 10 min in phase contrast or DIC and fluorescence modes using a CCD camera (Zeiss AxioCam MRm) or a Hamamatsu ORCA-flash4.0. The time resolution ensures reliable measurement of cell size and protein content changes, while minimizing cell damage.

## Supplementary Material

Appendix 01 (PDF)

Movie S1. Movies of cell growth, division, and death for example wt cells of phenotypes Ia, Ib, and II. The images are an overlay of DIC, the chromosome in green, and propidium iodide in red.

Movie S2. An example of a cell that loses its PRJ2001-GFP-Fis plasmid and undergoes lysis due to the antibiotics present in the media. This cell loses its plasmid at 54:15 hr, proceeds to divide seven additional times, becomes filamented, and undergoes lysis at 56:15 hr.

## Data Availability

Numerical data have been deposited in Zenodo (https://zenodo.org/records/11044898) (85).
