# Supplementary material for: Nonlethal deleterious mutation–induced stress accelerates bacterial aging
Source: Proc Natl Acad Sci U S A. 2024 May 6;121(20):e2316271121. doi: 10.1073/pnas.2316271121 (PMC11098108; doi:10.1073/pnas.2316271121)
Supplement: Supplementary file 1 — Appendix 01 (PDF) [file pnas.2316271121.sapp.pdf]

PNAS

Supporting Information for

Non-lethal, deleterious mutation-induced stress accelerates bacterial aging

Maryam Kohram, Amy E. Sanderson, Alicia Loui, Peyton V. Thompson, Harsh Vashistha, Aseel Shomar, Zoltán N. Oltvai, and Hanna Salman

Corresponding Authors: Zoltán N. Oltvai and Hanna Salman

Email: zoltan_oltvai@urmc.rochester.edu, hsalman@pitt.edu

This PDF file includes:

Supporting text
Figures S1 to S8
Tables S1 to S2
Legends for Movies S1 to S2
SI References

Other supporting materials for this manuscript include the following:

Movies S1 to S2

Supporting Information Text

1. Environment-dependent growth differences in deletion mutant strains

When grown in LB medium, some mutants with a deleted ATP synthase subunit (Δ*atpA*, Δ*atpD*, Δ*atpE*, Δ*atpF*, Δ*atpH*), a DNA repair and stress response related gene (Δ*recB*), ubiquinone biosynthesis related genes (Δ*ubiF*, Δ*ubiH*), a gene related to electron transport (Δ*nuoJ*), a tricarboxylic acid cycle related gene (Δ*sucB*), a bacteriocin transport gene (Δ*tolR*), and one other strain (Δ*fkpB*), were found to have significantly lower growth rates compared to wild-type (wt) *E. coli* (Figure S2B). However, of these strains, only six of them showed a significant lower saturation point compared to wt cells (Δ*atpA*, Δ*atpD*, Δ*atpH*, Δ*rseA*, Δ*sucB*, Δ*ubiF*,). When grown in M9CG or M9G media, a different set of mutants showed significantly lower growth rates. These strains consist of ATP synthase subunit deleted mutants (Δ*atpA,* Δ*atpD,* Δ*atpE,* and Δ*atpF*), mutants with deleted genes related to sulfur metabolism (Δ*cysC*, Δ*cysD*, Δ*cysE*, Δ*cysG*, Δ*cysH*, Δ*cysI*, Δ*cysN*, Δ*cysP*, Δ*cysU*, Δ*cysW*), and other mutants (Δ*rimM,* Δ*fkpB,* Δ*iscS,* Δ*lipB,* Δ*lpd,* Δ*nuoA,* Δ*nuoK,* Δ*recB,* Δ*ydjI,* Δ*ubiF,* Δ*ubiH,*). Seven of these strains (Δ*cysC*, Δ*cysD*, Δ*cysG*, Δ*cysH*, Δ*cysI*, Δ*cysN*, and Δ*cysU*), demonstrated a lower saturation point than wt *E. coli* cells in M9CG. Also, all of these mutants in addition to one more strain (Δ*cysE*) exhibited a significantly lower SPG when grown in M9G. One strain had lower growth rate than wt in M9CG, but higher in M9G (Δ*ydjI*). As expected, all values for growth rate are lower than wt (except for the two cases of Δ*degP* and Δ*ydjI* in M9G), illustrating that no single gene deletion increases the normal growth rate of *E. coli* cells in these experimental conditions. It is also notable that some mutants have a closer growth rate value to wt *E. coli* in M9CG than in LB; for example, Δ*atpA* exhibits a more similar growth to wt *E. coli* when in M9CG, and even more similar when grown in the nutrient limited medium, M9G.

2. Gene expression profiles in deletion mutant strains

The *E. coli* strain BW25113 harbors a complex genetic network comprising of 4,487 genes and 459 pathways[1], which are responsible for carrying out various biological processes. In this section, we aimed to investigate the response of single gene deleted mutants to stress induced by nutrient limitation. To this end, we analyzed and compared the gene expression profiles of relevant genes of ATP subunit deleted mutants and the wt strain under three different media conditions. Principal component analysis was performed separately within each growth medium, and the first two principal components are presented in Figure S4A. Distinct clustering patterns are evident across the three media conditions, emphasizing significant transcriptional differences between these two strains.

Previous studies have identified genes that become essential when cells were grown in nutrient limited conditions[2]. Figure S4B illustrates the expression profiles for 124 of these genes that were grouped according to their involvement in metabolic pathways[3]. Notably, significant alterations were observed primarily in genes associated with central metabolism, as well as those related to vitamin and cofactor metabolism pathways. These alterations in gene expression likely reflect a cellular response aimed at optimizing resource allocation, rerouting metabolic pathways, and enhancing nutrient uptake and utilization, thus triggering the allostasis response. The

identification of these genes provides insight into the regulatory networks that enable cells to maintain essential metabolic functions and survival under challenging nutritional conditions.

Furthermore, to investigate how stress response in single cells can lead to different cellular morphologies, we examined the genes related to filamentation and the SOS response in *E. coli* cells. *sulA*, *minCD*, and *ftsZ* are key genes that play essential roles in the filamentation process[4,5]. Figure S4C displays the expression profiles of these genes, along with other filamentation-associated genes identified as essential[6,7]. The observed downregulation in the expression of *ftsZ*, which is vital for Z-ring formation and cell division regulation, as well as the upregulation of *minE* expression, known to restrict Z-ring formation, under nutrient-limited conditions, agrees with the increase in filamentation observed in our single cell experiments. This concurrence between gene expression changes and the alteration in death phenotypes supports the notion that stress conditions can trigger shifts in cell death phenotypes rising from the adaptive mechanisms underlying allostasis.

3. Probabilities of single cell death and their impact on population growth

Many mathematical models have been proposed for describing bacterial population growth; however, most do not take single cell behavior into consideration[8]. Among these models, empirical analytical equations such as the logistic model are commonly used. In our study, we observed that our population growth curves (from Figure S1) were well-fitted by a logistic model represented by the equation $N(t) = \frac{N_0 \frac{k}{\lambda}}{N_0 + \left(\frac{k}{\lambda} - N_0\right) \exp(-kt)}$, where N represents the population number as a function of time, t, N_0 is the initial cell count, k is the growth rate coefficient, and λ is the suppression rate coefficient[9]. Figure S7A demonstrates a good fit of our data to this model. The parameter λ is typically associated with growth suppression resulting from nutrient limitation. Here, we aim to clarify whether the growth suppression could be attributed to a combination of growth arrest and death due to bacterial aging rather than due to nutrient limitation.

We propose a model that integrates the results of our single cell experiments to predict bacterial death and growth arrest associated with aging. The model is based on two of our key findings: (1) the occurrence of a post-replicative lifespan preceding cell lysis, and (2) the survival functions shown in Figure 5E. The underlying principles of our model are presented in Fig. S7B. Each time-step, denoted as g, represents the generation time, which is considered to be constant for simplicity. The fate of each cell is independent of other cells. Within each time-step, a cell faces two possibilities: it can either be in its replicative lifespan and divide with probability $P_R(g)$, or it can enter its post-replicative lifespan with probability $P_{PR}(g)$. In the event of cell division, the same two options are presented in the subsequent time-step ($P_R(g+1)$ and $P_{PR}(g+1)$). Conversely, if the cell enters its post-replicative lifespan, it encounters two new options in the next time-step: either it remains in its current state with a probability $P_{PR}(g+1)$, or it undergoes cell death with a probability $P_D(g+1)$. The values of these probabilities vary across strains and media conditions and are determined based on the survival functions presented in Figure 5E. The red lines in these graphs represent fitted Gompertz distributions, which serve as the basis for acquiring the probability distributions presented in Figure S7C. Let $S_1(t)$ be the survival function

for lysed cells (Figure 5E), and $S_2(t)$ be the survival function for replicating cells (Figure S6D). By using these functions, we can find the three probabilities as follows: $P_{PR}(t) = S_2(t)$, $P_D(t) = 1 - S_1(t)$, and $P_R(t) = S_1(t) - S_2(t)$.

Figure S7C presents the outcomes of our model, revealing that bacterial aging has an influence on the population growth experiments conducted in this study. A previous study has investigated single-cell behavior during population growth and introduced three probabilities for individual cells: division, death, or remaining alive but non-dividing[10]. These probabilities are similar to the concepts we have introduced in our model, with the difference that they were attributed to nutrient depletion during population growth. However, other studies have demonstrated that under nutrient-depleted conditions, cells enter a state of growth arrest rather than undergo cell death[11]. Here, we hypothesize that during population growth, cells undergo these three fates, which occur due to aging. This study reveals that nutrient limitation accelerates the aging process, as evidenced by the survival functions in Figure 5E. Particularly in microbatch experiments, where nutrient depletion occurs rapidly, the aging rate of bacteria can undergo rapid changes due to nutrient scarcity. Hence, the parameter λ in the logistic model could be attributed to aging due to nutrient limitation. The precise mechanism by which nutrient depletion affects the aging rates needs further investigation.

4. Data analysis

Microbatch culture data analysis

In a previous study, growth of non-lethal *E. coli* isogenic single-gene knockout mutants (the Keio collection) on semi solid agar was investigated, and three *E. coli* colony growth values – lag time of growth (LTG), maximum growth rate (MGR) and saturation point of growth (SPG) – were measured using a high-throughput measurement system. The results were able to identify a subset of genes whose deletion delayed colony formation or changed saturation point and presented a better understanding of the range of phenotypes that exist in this strain[12]. We chose 65 mutants among this subset which are listed in Table S1, and measured their MGR and SPG, as explained below. We did not measure LTG for our microbatch culture experiments since the lag time depends on many initial condition factors that are not related to the interests of this study. However, we measured the Area Under Curve (AUC) and time of exponential phase (T_{exp}) instead.

To determine the maximum growth rate (MGR) and saturation point of growth (SPG) for each mutant, we employed the following procedure. First, any OD_{600} values below zero were adjusted to zero, as negative values lack meaningful interpretation and result from measurement errors in low concentrations. Subsequently, background measurements obtained from blank samples, where only the growth medium was present in the well, were subtracted from each population growth curve on a point-by-point basis. To identify the MGR, we located the maximum point of the first derivative of $ln\left(\frac{N}{N_0}\right)$, and then selected five points preceding and following this maximum. A linear regression was fitted to these eleven points, and the slope of the line provided the MGR. For SPG determination, the last 20-50 points of the growth curve were forced to fit a horizontal line, and the value of this line was recorded as the SPG.

To measure AUC and T_{exp}, we applied methods described in a previous study[13]. We determined the AUC by using MATLAB's trapezoid integration function with time intervals set at 0.1 hours. T_{exp} was calculated by first applying a piecewise linear fit to $ln\left(\frac{N}{N_0}\right)$ to find the exponential phase. Subsequently, we determined the duration during which the graph exhibited exponential growth.

A demonstration of these calculations for a sample growth curve (wild-type in LB) is presented in Figure S2A. The extracted values from these calculations are presented in Figure S2B-C.

Data reduction and clustering

We calculated the Pearson correlation coefficients between the parameters (Figure S2D), which revealed strong correlations among these variables. This suggests the possibility of reducing the dimensionality of the dataset. To extract the underlying dimensions characterizing the growth curves of microbatch cultures, we performed principal component analysis (PCA). All the parameters (MGRs, SPGs, AUCs and T_{exp}s) distinguishing the different growth curves of all 65 mutants + 1 wild-type strain across three different media conditions form a 66×12 matrix, which we label X. Using the built-in MATLAB function "pca", we computed the principal components of this matrix. The scree plot displayed in Figure S2E indicates that more than 95% of the variance can be explained by the first two principal components, justifying a dimension reduction to two. The projection of the entire dataset onto these two principal components is presented in Figure 1A. Figure S2F demonstrates the most relevant parameters to the first two principal components. For clustering analysis, we utilized the hierarchical method and the built-in MATLAB function "clusterdata", with the maximum number of clusters set to two.

Microarray Analysis

Raw Affymetrix CEL files (E. coli Genome 2.0) were processed with Expression Console (version 1.4.1.46), using the Robust Multiarray Average (RMA) to produce normalized probeset-level expression values and the MicroArray Suite 5.0 (MAS5) algorithm to produce Absent/Present calls for each probeset in each sample. Array quality was also assessed by computing Relative Log Expression (RLE) and Normalized Unscaled Standard Error (NUSE) values using the affy (version 1.70.0) and affyPLM (version 1.68.0) R packages. Principal Component Analysis (PCA) was performed using the prcomp R function with expression values that had been normalized across all samples to a mean of zero and a standard deviation of one. All analyses were performed using the R environment for statistical computing (version 4.1.2).

Single-cell data analysis

The movies acquired from the mother machine experiments were manually analyzed to count the number of dead cells, time of death, percentage of death phenotypes, and number of generations presented in Table S2 and the data points in Figure 5E and S6D.

We note that during the experiment, a subset of cells loses its plasmids and dies as a result of losing the antibiotic resistance gene contained in that plasmid (last column of Table S2). This

mode of death was identified by a gradual decrease in the GFP-Fis fluorescence in the cell, while the cell continued to divide (Movie S2). GFP-Fis is produced from the antibiotic resistance-carrying plasmid in the cell. Its sudden loss would lead to a halt in GFP-Fis production and consequently a gradual decrease in the fluorescence intensity in the cell as the cell depletes the protein during subsequent division events. Since the death of such cells is due to our experimental conditions, we did not consider them in our analysis of death phenotypes.

The fits in Figures 5E, S6D, and S8C were performed in MATLAB to the equation $y = exp\left(-\frac{a}{b}\left(e^{bt}-1\right)\right)$ where a is a constant, b is the aging rate, and t is time, using a standard linear least squares regression method. The values for aging rate reported throughout the entire study are the values that were acquired for b using this fit.

The software Oufti[14], along with custom designed MATLAB codes were used to measure the length of the mother cell. An example output of these measurements along with an explanation of parameter extraction is provided in Figure S6B. The values for exponential elongation rate (EER) and birth length (BL) for each generation were extracted from fitting the data points of each generation to the equation $x_n(t) = x_n(0)e^{\alpha_n t}$, where x is length, n is generation number, t is time, and α is EER. Doubling rate (DR) was determined by reversing the generation time, $DR = \frac{1}{T_n}$, where T_n is the generation time. In cases where the individual cell divided one last time and the daughter did not grow in size (which means the last cycle had an exponential elongation rate of zero), generation zero was considered to be the cycle preceding the last division.

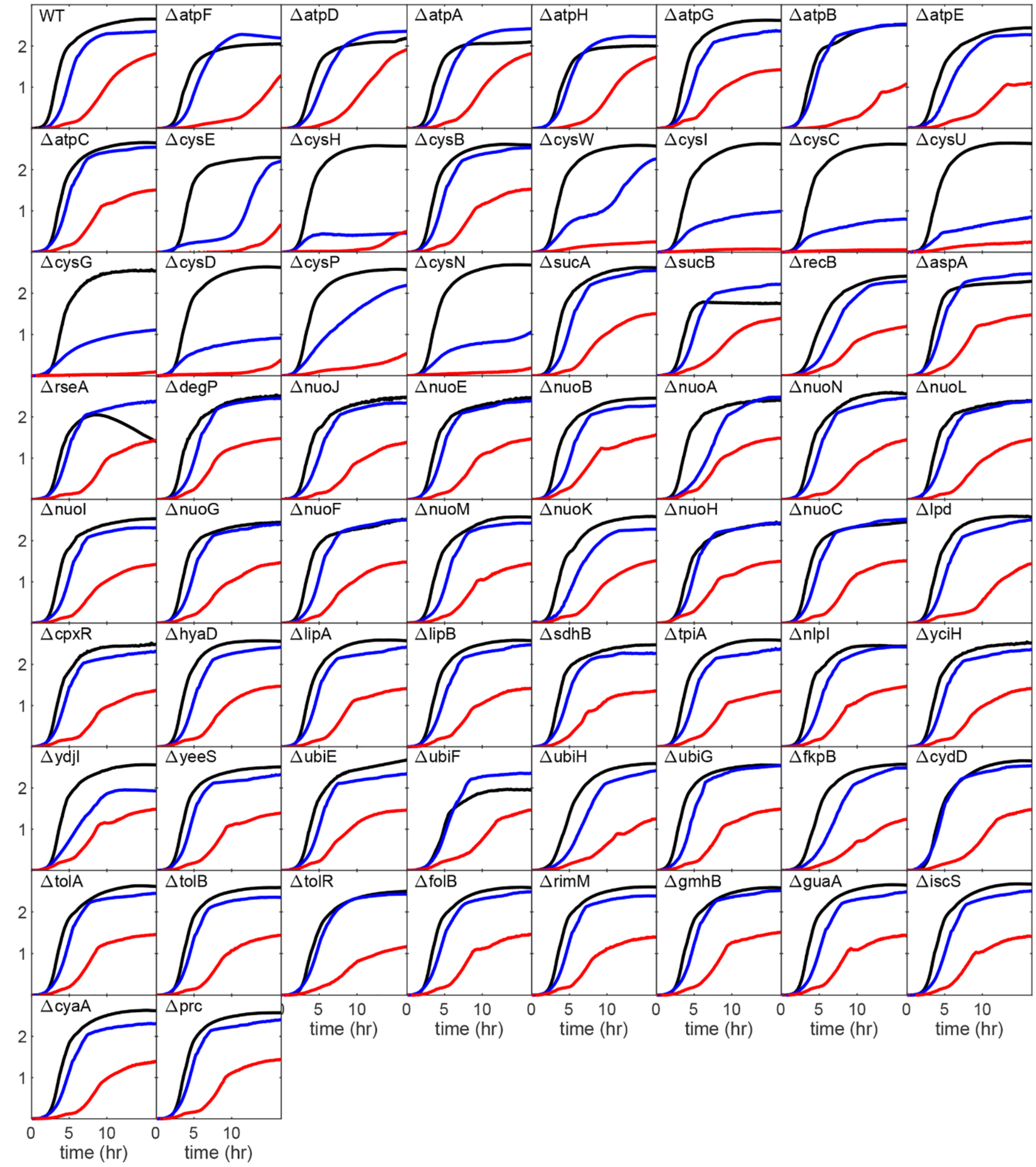

Fig. S1. Population growth in microbatch cultures

Growth curves for wild-type *E. coli* and its 65 tested isogenic mutants in LB (black), M9CG (blue) and M9G (red) growth media are shown. The y-axis is $\ln\left(\frac{N}{N_0}\right)$, where N is OD_{600} measurements and N_0 is the initial OD_{600} values. The decline of some growth curves after reaching saturation indicates a death phase of the population.

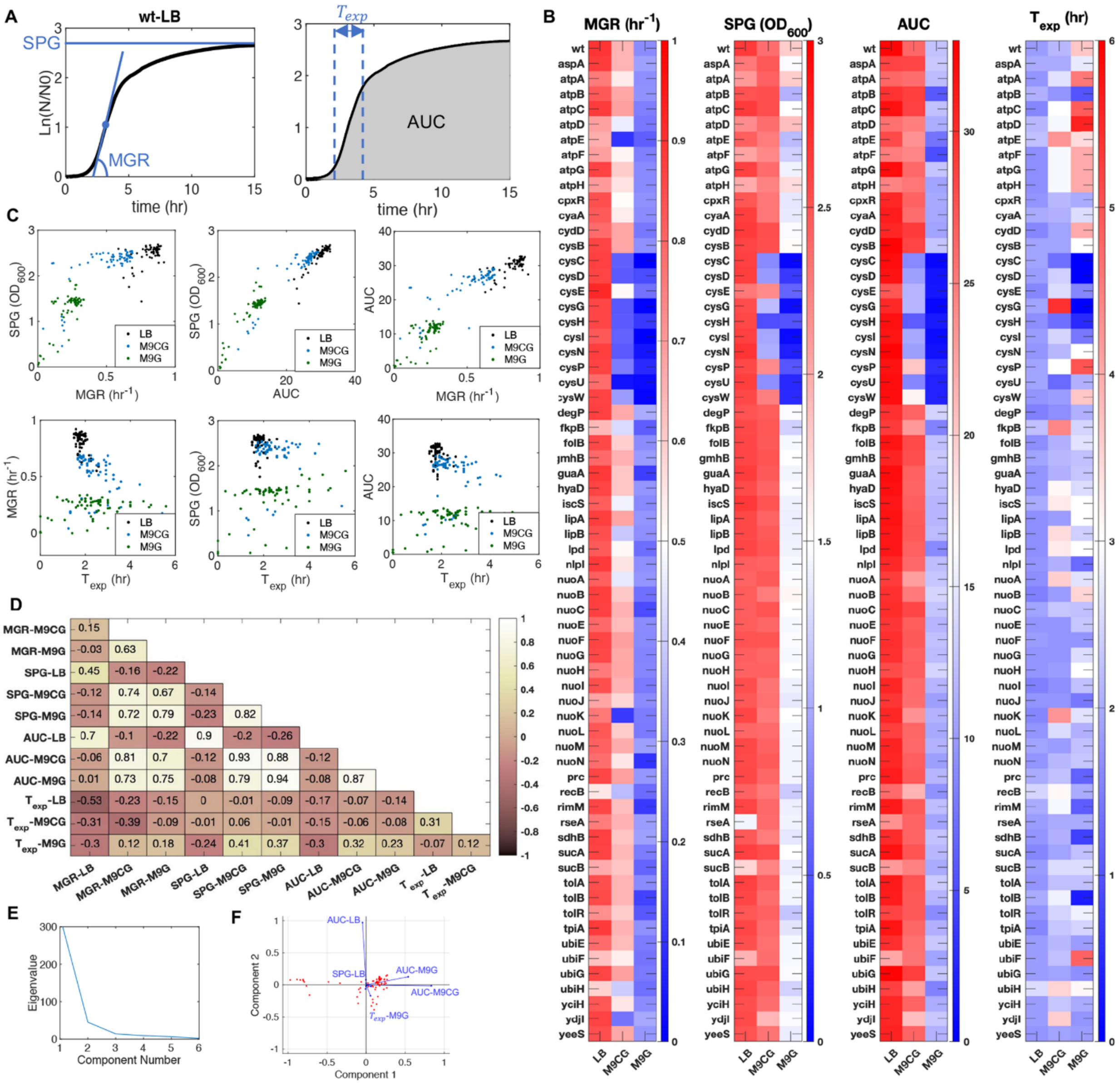

Fig. S2. Growth parameters of wt and mutant *E. coli* strains in microbatch cultures

(A) The Maximum Growth Rate (MGR), Saturation Point of Growth (SPG), Area Under Curve (AUC), and time of exponential phase (T_{exp}) were extracted from the growth curves using established methods. MGR was calculated as the maximum slope of a fitted line from 5 points before and 5 points after the maximum of the first derivative of $ln(\frac{N}{N_0})$, where N is OD_{600} measurement and N_0 is the initial OD_{600} value. For wt *E. coli* in LB, the MGR was $0.89\ hr^{-1}$. SPG was determined by fitting the last 20-50 points of the growth curve to a straight horizontal line. For wt *E.coli* in LB medium the SPG was 2.65. AUC was determined by integrating the growth curve. For wt *E. coli* in LB medium the AUC was 32.51. T_{exp} was found by using a piecewise linear fitting method and finding the time in which the growth is completely exponential. For wt *E. coli* in LB medium the T_{exp} was $1.83\ hr$. (B) The MGR, SPG, AUC, and T_{exp} values that were extracted using the above method for all 65 single-gene mutations presented in Figure S1 are shown here

in a color-coded format, colors present the values of the parameters. (C) Plots of MGR against SPG, and AUC against MGR and SPG values reveal high correlations between the variables justifying a reduction in the number of parameters and principal component analysis. (D) Pearson correlation coefficients were calculated for all parameters extracted from the growth curves in three different environments for 65 *E. coli* single gene deletion mutants and the wt. Some of the correlations are significant justifying a principal component analysis. (E) Cumulative sums of eigenvalues were calculated to determine the percentage of variance explained by each principal component. The first two components explain approximately 95% of the variance of the data. (F) The orthonormal principal component coefficients for each variable and the principal component scores are visualized showing that the most relevant parameters for PC1 are AUC-M9CG and AUC-M9G and for PC2 are AUC-LB, T_{exp}-M9G and SPG-LB.

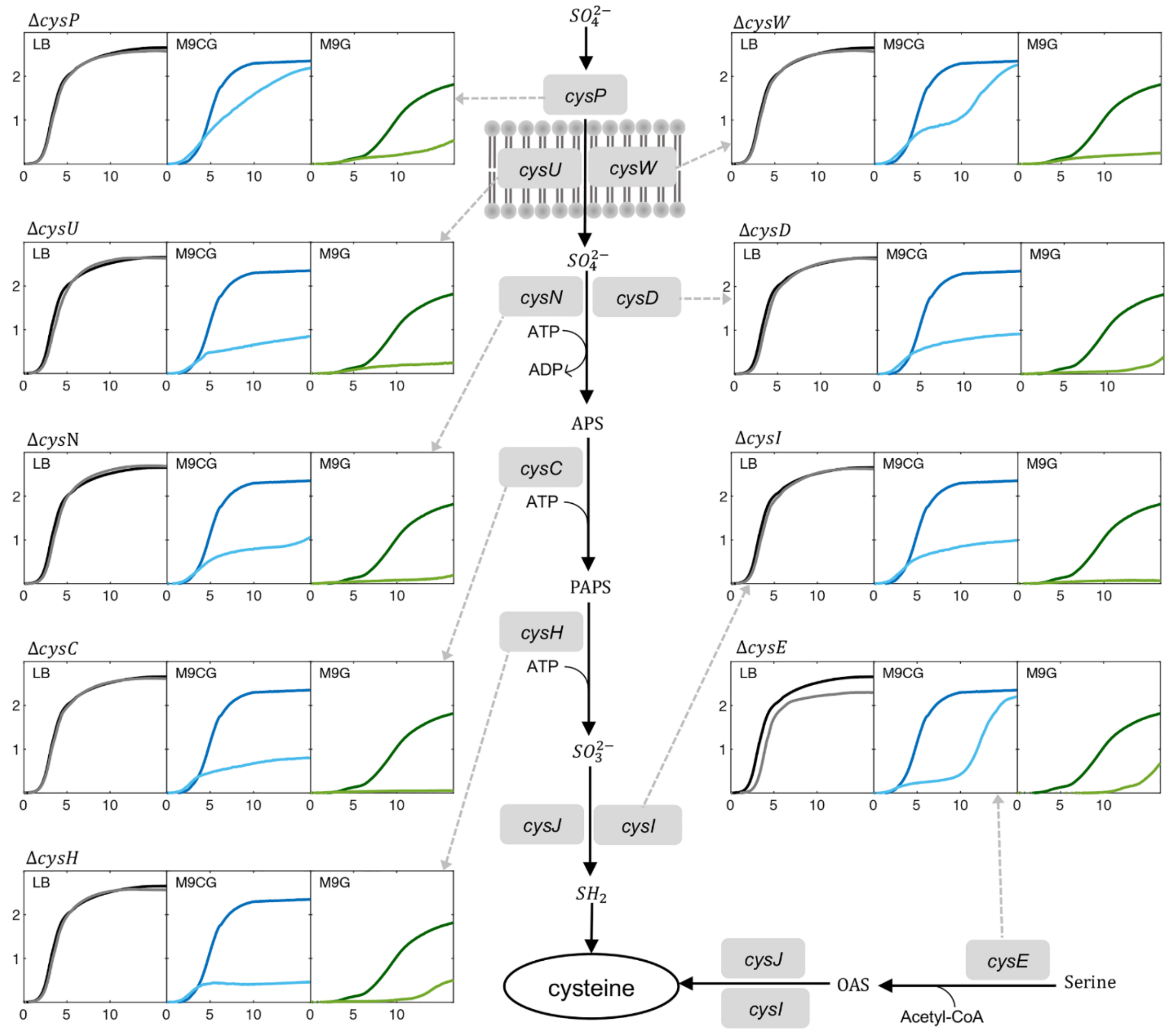

Fig. S3. Cysteine metabolism pathway and its enzymes in *E. coli*

The growth of wt (darker lines) and single-gene subunit deletion mutants (lighter lines) are displayed in graphs surrounding the cysteine metabolism pathway and its enzymes. Seven single deletion mutant strains ($\Delta cysC$, $\Delta cysD$, $\Delta cysE$, $\Delta cysH$, $\Delta cysI$, $\Delta cysN$, $\Delta cysU$) belonging to cluster I from Figure 1A, display slow or no growth in nutrient-limited conditions (M9CG and M9G). The other two mutants ($\Delta cysP, \Delta cysW$) belong to cluster II and display adaptive behaviors in M9CG. The y-axis is $\ln\left(\frac{N}{N_0}\right)$, where N is OD_{600} measurements and N_0 is the initial OD_{600} values, and x-axis is time (hours).

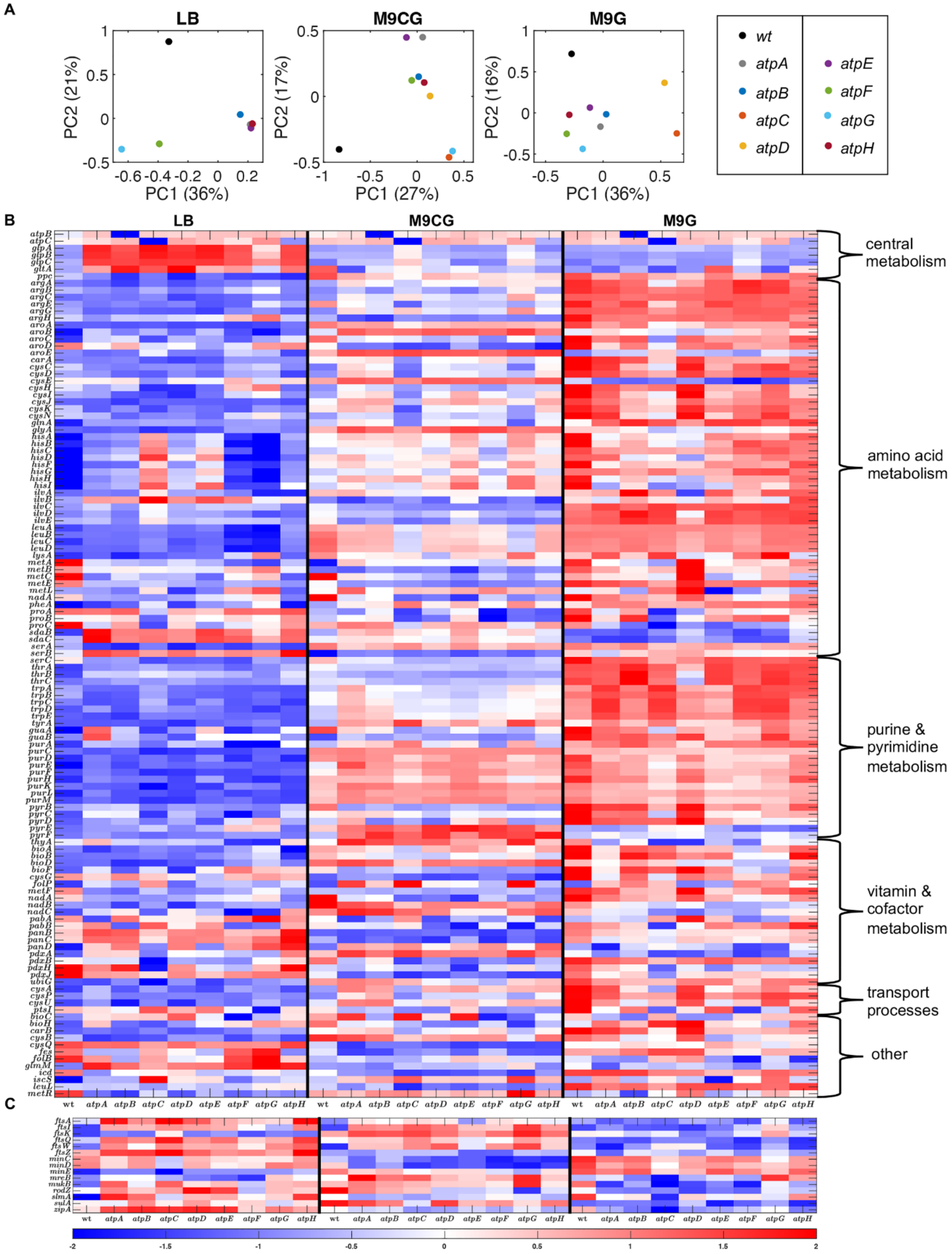
A
LB
M9CG
M9G
PC1 (36%)
PC2 (21%)
PC1 (27%)
PC2 (17%)
PC1 (36%)
PC2 (16%)
wt
atpA
atpB
atpC
atpD
atpE
atpF
atpG
atpH
B
LB
M9CG
M9G
central metabolism
amino acid metabolism
purine & pyrimidine metabolism
vitamin & cofactor metabolism
transport processes
other
C
-2
-1.5
-1
-0.5
0
0.5
1
1.5
2

Fig. S4. Gene expression profiles across environmental conditions

(A) Principal component analysis was employed to assess the transcriptome dataset under specific media conditions. The values of the first two principal components are presented for each environmental condition, providing an overview of gene expressions in different media. (B) Expression profiles of those 124 genes that become essential when cells are exposed to nutrient-limited conditions and experience stress[2]. These genes are categorized based on their involvement in distinct metabolic pathways, highlighting their functional relevance in response to nutrient limitation-induced stress. (C) Expression profiles of 15 genes essential for filamentation[4–7]. These expressions align with the observation of an increase in the occurrence of filamentation in the last cell cycle (phenotype II) under nutrient-induced stress.

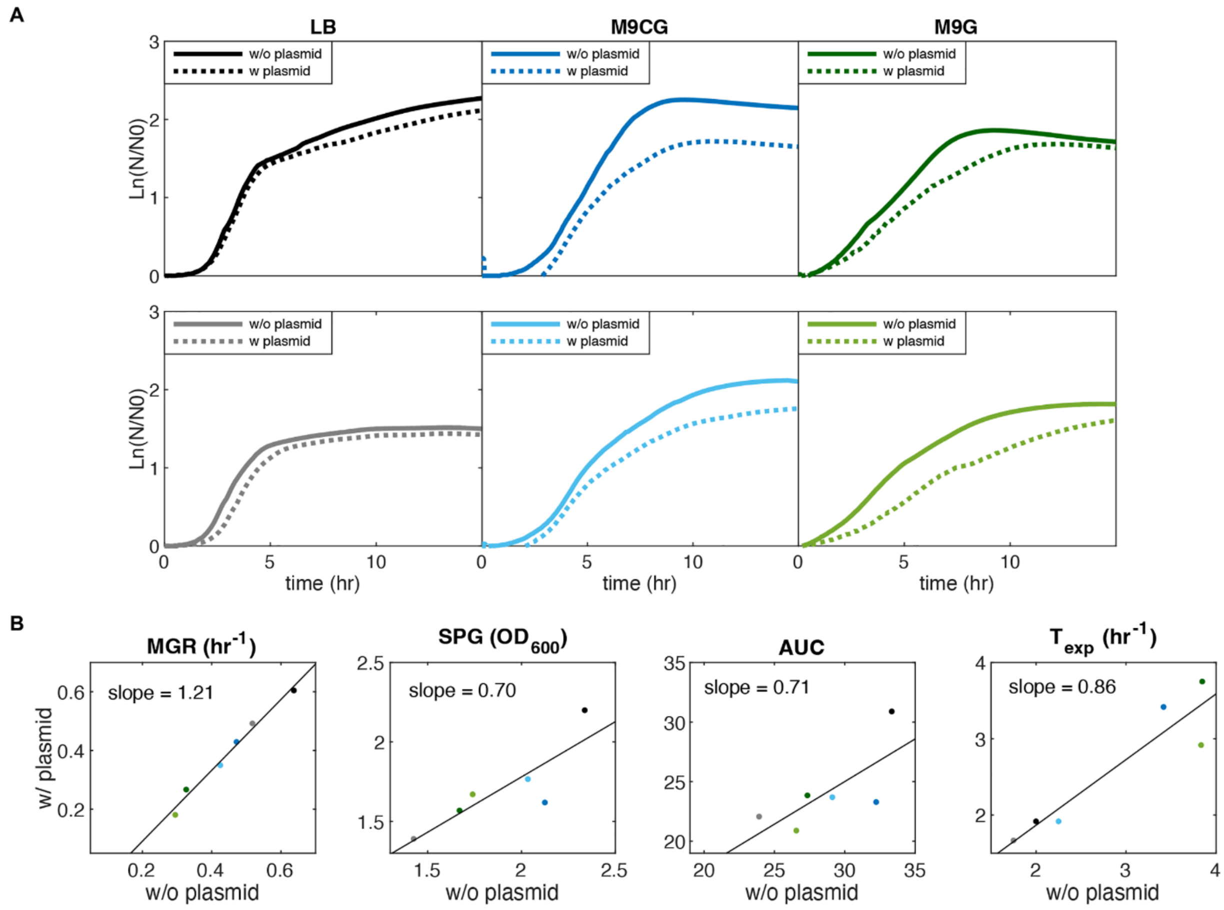

Fig. S5. Comparison of the growth curves for plasmids-containing and plasmids-lacking strains

(A) A comparison of the growth curves of the two strain types investigated in this study. In the microbatch experiments, cells did not contain any plasmids (w/o plasmid), while in the mother machine experiments, cells contained two plasmids (w/ plasmid), namely pZA3R-mcherry and pRJ2001-GFP-Fis (see Materials and Methods for details). Y-axis is $ln(\frac{N}{N_0})$, where N is OD_{600} measurement and N_0 is the initial OD_{600} value. (B) A comparison of the MGR, SPG, AUC, and T_{exp} of the two strains. All growth parameters are linearly proportional to each other. The line represents a linear regression of the data points, with the slope value depicted in the plot.

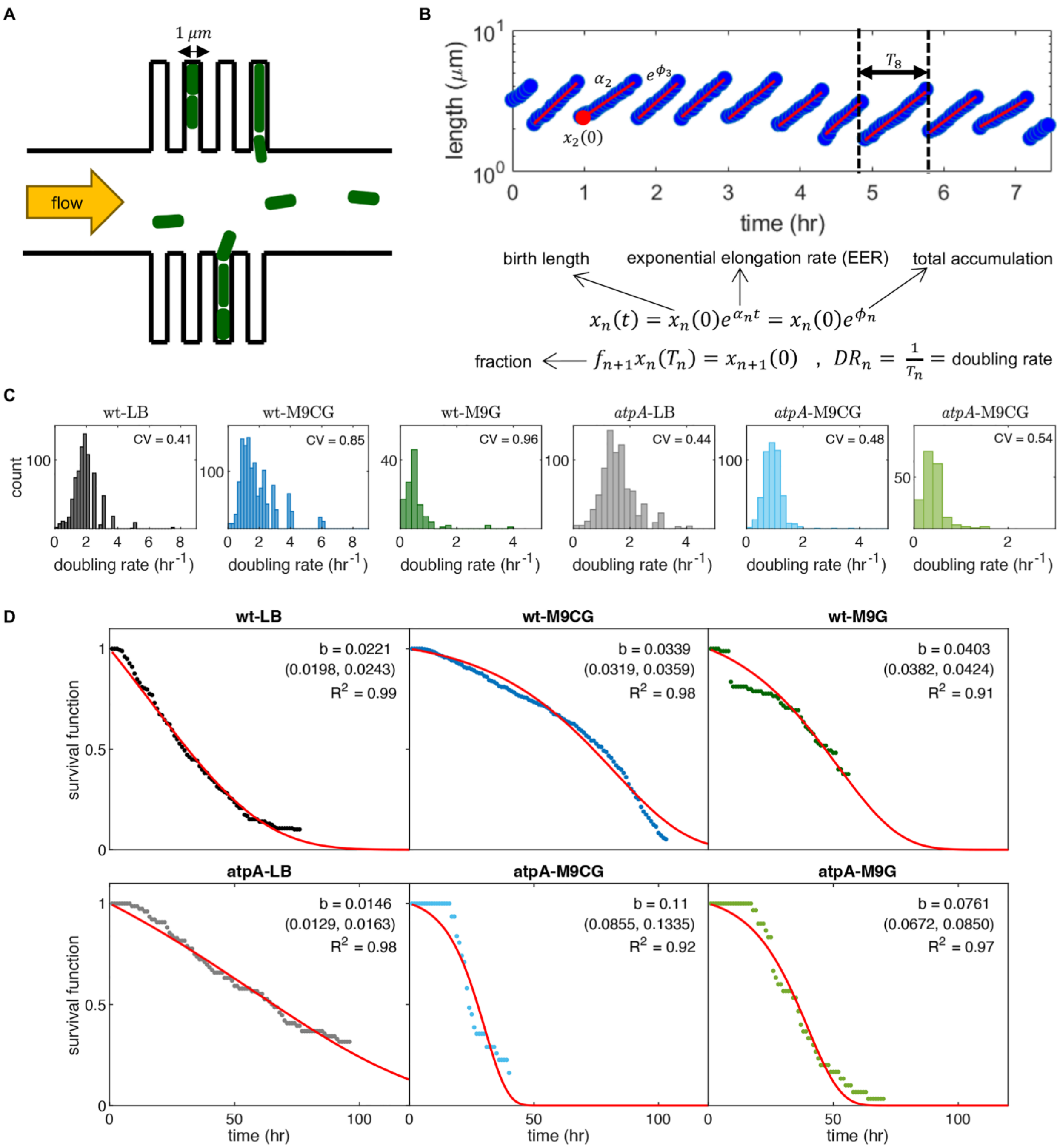

Fig. S6. Microfluidic setup and lifespan distributions of *E. coli* strains

(A) The mother machine was used to track individual bacterial cells for multiple generations until they died. This microfluidic device contains microchannels of similar width of a single wt *E. coli* cell grown in LB ($\sim 1\ \mu m$) and a length sufficient for multiple cells ($\sim 30\ \mu m$), with a larger channel (width and height of $\sim 30\ \mu m$) for continuous media flow to support the growth of trapped cells and wash away daughter cells. (B) Example of cell length measurements over generations, with blue dots representing measurements and red lines showing exponential fits to the equation $x_n(t) = x_n(0)e^{\alpha_n t}$, where x is cell length, t is time, n is generation number, and α is the exponential

elongation rate (EER). (C) Distributions of single-cell wt and $\Delta atpA$ doubling rates in three different media. CV is coefficient of variation = $\frac{\text{standard deviation}}{\text{mean}}$. (D) Survival functions of wt and $\Delta atpA$ in three media conditions, where only the aRL of cells are considered as their lifetime. Dotted lines represent experimental data, and red lines are a fit to a Gompretz equation, which allows for estimation of the aging rate ($b\ (hr^{-1})$) shown on each graph. The numbers in the parentheses present 95% confidence bounds.

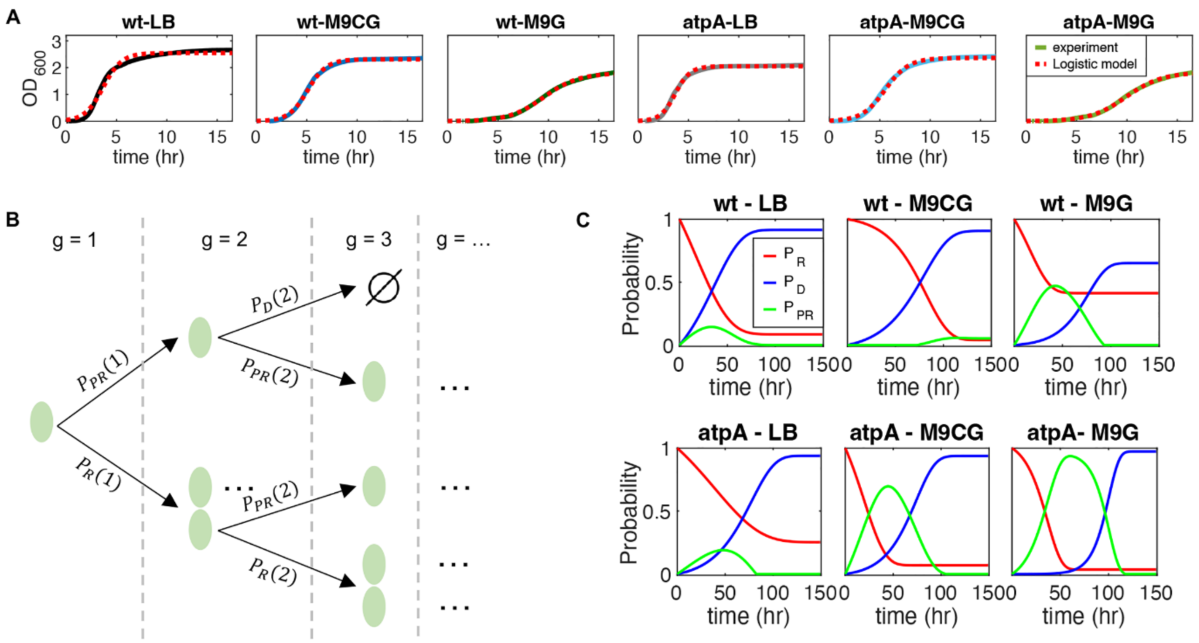

Fig. S7. Aging probabilities of *E. coli* determined from single-cell experiments

(A) The population growth curves exhibit a good fit to the logistic model. Solid lines represent experimental data and dotted lines depict the logistic model fit. (B) Starting at generation one (g = 1), the cell has two options. With probability $P_{PR}(g=1)$ it will enter its post-replicative lifespan, and with probability $P_R(g=1)$ the cell will divide into two daughter cells. If the mother cell divides, the two daughter cells that are now in generation two (g = 2), will have the same possible fates, but with new probabilities ($P_{PR}(g=2)$ and $P_R(g=2)$). If the cell enters its PRL, it will have the probabilities to either die in the next generation ($P_D(g=2)$) or stay in its post-replicative lifespan ($P_{PR}(g=2)$). (C) Probability functions in all conditions. At the beginning of a cell's lifespan, the cell has a high probability to divide, and a low probability to die or enter its post-replicative lifespan. The probability to enter PRL increases during the middle of its lifespan, and the probability of death increases during the end of its lifespan.

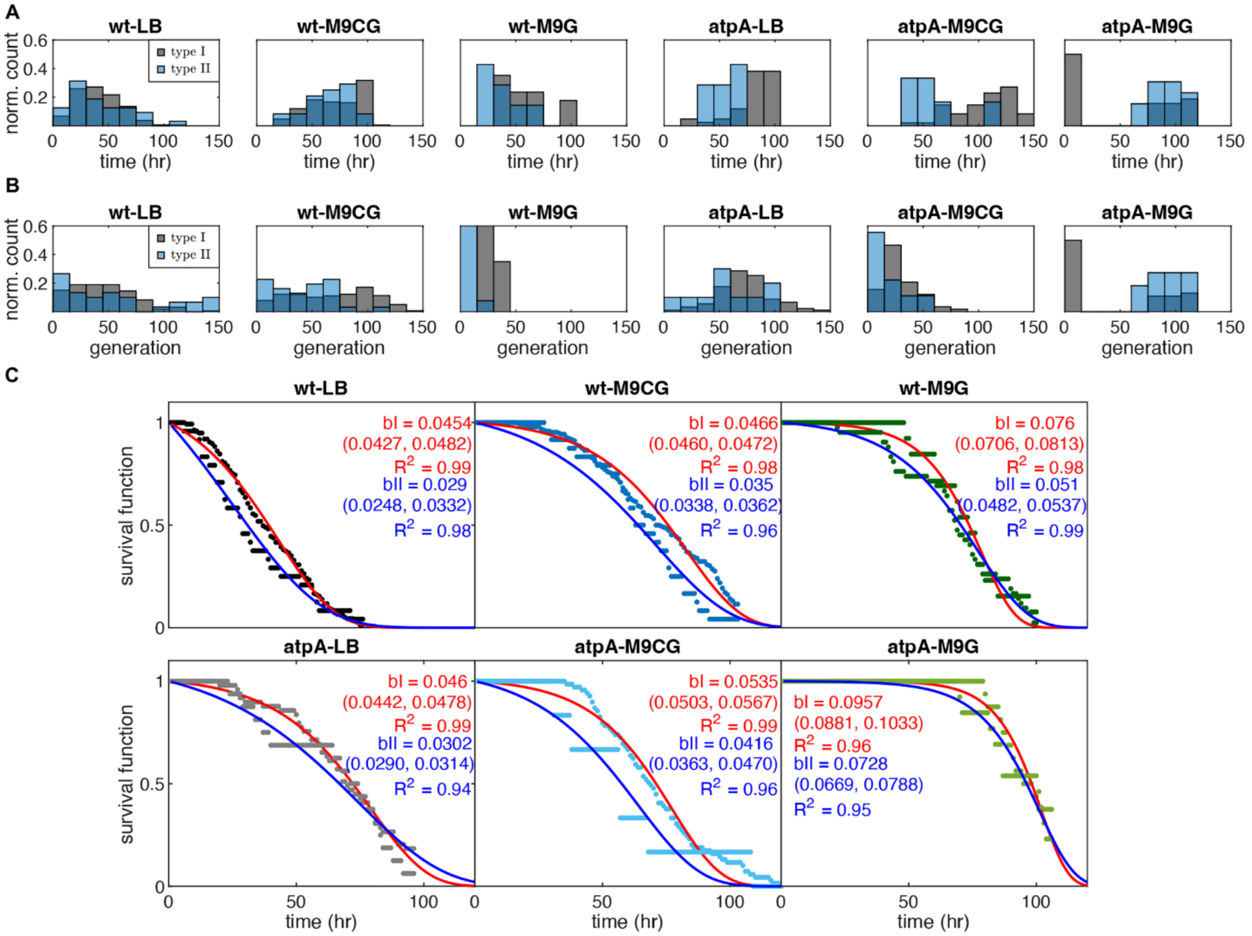

Fig. S8. Distribution of lifespans in terms of number of generations and survival functions for different phenotypes in three different media

(A) The entire lifespan (aRL + PRL) of each phenotype is represented for two strains (wt and *ΔatpA*). (B) The apparent replicative lifespan (aRL) of each phenotype is represented for two strains (wt and *ΔatpA*) in terms of the number of generations. (C) Survival functions of wild-type and *ΔatpA* strains are divided into two phenotypes. The dotted lines are the experimental data, the solid lines are fits to a Gompertz equation, where $b(hr^{-1})$ is the aging rate presented on the graph and the numbers in the parentheses are 95% confidence bounds. Red represents phenotype I and blue represents phenotype II. To account for the uncertainty of whether the surviving cells at the end of the experiment are phenotype I or II, we excluded them from the analysis, resulting in all survival functions to go to zero at the experiment's endpoint.

Table S1. List of isogenic single-gene-deletion *E. coli* K-12 strain BW25113 mutants used in this study. All strains were obtained from the Keio collection.[15]

Deleted Gene	location, protein, subunit
aspA	cytosol, aspartate ammonia-lyase
atpA	inner membrane, ATP synthase; F1 sector; alpha-subunit
atpB	inner membrane, ATP synthase; F0 sector; subunit a
atpC	inner membrane, ATP synthase; F1 sector; epsilon-subunit
atpD	inner membrane, ATP synthase; F1 sector; beta-subunit
atpE	inner membrane, ATP synthase; F0 sector; subunit c
atpF	inner membrane, ATP synthase; F0 sector; subunit b
atpG	inner membrane, ATP synthase; F1 sector; gamma-subunit
atpH	inner membrane, ATP synthase; F1 sector; delta-subunit
cpxR	cytosol, DNA-binding transcriptional dual regulator CpxR
cydD	inner membrane, glutathione/L-cysteine ABC exporter subunit CydD
cysB	cytosol, DNA-binding transcriptional dual regulator CysB
cysC	cytosol, adenylyl-sulfate kinase
cysD	cytosol, sulfate adenylyltransferase subunit 2
cysE	cytosol, serine acetyltransferase
cysG	cytosol, siroheme synthase
cysH	cytosol, phosphoadenosine phosphosulfate reductase
cysI	sulfite reductase, hemoprotein subunit
cysN	cytosol, sulfate adenylyltransferase subunit 1
cysP	periplasmic space, thiosulfate/sulfate ABC transporter periplasmic binding protein CysP
cysU	inner membrane, sulfate/thiosulfate ABC transporter inner membrane subunit CysU
cysW	inner membrane, sulfate/thiosulfate ABC transporter inner membrane subunit CysW
degP	inner membrane, periplasmic space, periplasmic serine endoprotease DegP
fkpB	cytosol, peptidyl-prolyl cis-trans isomerase FkpB
folB	cytosol, dihydroneopterin aldolase
gmhB	cytosol, D-sedoheptulose 7-phosphate isomerase; GDP-heptose biosynthesis; T-phage resistance
guaA	cytosol, GMP synthetase
hyaD	cytosol, putative hydrogenase 1 maturation protease HyaD
iscS	cytosol, cysteine desulfurase
lipA	cytosol, lipoyl synthase
lipB	cytosol, lipoyl(octanoyl) transferase
lpd	inner membrane, cytosol, lipoamide dehydrogenase
nlpI	inner membrane, outer membrane, lipoprotein NlpI
nuoA	inner membrane, NADH:quinone oxidoreductase subunit A
nuoB	inner membrane, NADH:quinone oxidoreductase subunit B

nuoC	inner membrane, NADH:quinone oxidoreductase subunit CD
nuoE	inner membrane, NADH:quinone oxidoreductase subunit E
nuoF	inner membrane, NADH:quinone oxidoreductase subunit F
nuoG	inner membrane, NADH:quinone oxidoreductase subunit G
nuoH	inner membrane, NADH:quinone oxidoreductase subunit H
nuoI	inner membrane, NADH:quinone oxidoreductase subunit I
nuoJ	inner membrane, NADH:quinone oxidoreductase subunit J
nuoK	inner membrane, NADH:quinone oxidoreductase subunit K
nuoL	inner membrane, NADH:quinone oxidoreductase subunit L
nuoM	inner membrane, NADH:quinone oxidoreductase subunit M
nuoN	inner membrane, NADH:quinone oxidoreductase subunit N
prc	periplasmic space, inner membrane, tail-specific protease
recB	cytosol, exodeoxyribonuclease V subunit RecB
rimM	cytosol, ribosome, ribosome maturation factor RimM
rseA	inner membrane, anti-sigma-E factor RseA
sdhB	inner membrane, succinate:quinone oxidoreductase, iron-sulfur cluster binding protein
sucA	cytosol, dihydrolipoyltranssuccinylase
sucB	cytosol, dihydrolipoyltranssuccinylase
tolA	inner membrane, Tol-Pal system protein TolA
tolB	periplasmic space,Tol-Pal system periplasmic protein TolB
tolR	inner membrane, Tol-Pal system protein TolR
tpiA	cytosol, triose-phosphate isomerase
ubiE	bifunctional 2-octaprenyl-6-methoxy-1,4-benzoquinone methylase and S-adenosylmethionine:2-DMK methyltransferase
ubiF	cytosol, 2-octaprenyl-3-methyl-6-methoxy-1,4-benzoquinol hydroxylase
ubiG	inner membrane, cytosol, bifunctional 3-demethylubiquinone-8 3-O-methyltransferase and 2-octaprenyl-6-hydroxyphenol methylase
ubiH	cytosol, 2-octaprenyl-6-methoxyphenol to 2-octaprenyl-6-methoxy-1;4-benzoquinone
yciH	cytosol, putative translation factor
ydjI	cytosol, putative aldolase YdjI
yeeS	cytosol, CP4-44 prophage; RadC-like JAB domain-containing protein YeeS

Table S2. Filamentation and death phenotype ratios of individual Δ*atpA* and wt cells grown in three growth media (LB, M9CG and M9G).

Exp. #. indicates individual experiments. The total number of cells in this table indicates how many cells were analyzed to determine the percentage of the phenotypes. This value might be different of that presented in Figure 5A since some cells were not followed until the end of PRL and could not be included in that dataset. Some experiments did not include the GFP-Fis plasmid, hence it was not possible to differentiate between type Ia and type Ib. The percentage of non-filamented cells are reported as one single number for type I for those experiments. The dashed lines in the last column are experiments where the cells did not contain the GFP-Fis plasmid. For an example of a cell losing its plasmid see Movie S2.

		exp. #	exp. time (hrs)	# of cells	% of non-proliferating cells	Type I cells %		Type II cells %	Aging rate (hr^{-1})	% of cells that lost GFP-Fis plasmid
						Ia	Ib			
wild-type	LB	1	122	85	77.6	77.6		22.4	0.02649	-
		2	84	185	91.3	7.3	78.2	14.5	0.03569	2.4
	M9CG	1	161	68	95.2	81.7		18.3	0.01399	-
		2	104	629	96.0	17.5	75.7	6.8	0.03814	2.3
	M9G	1	58	79	64.6	39.2		60.8	0.05277	-
		2	50	85	26.0	31.8		68.2	0.09208	-
Δ*atpA*	LB	1	95	109	96.3	90.5		9.5	0.0463	-
		2	65	223	90.1	0	81.1	18.9	0.0546	1.6
	M9CG	1	140	99	98	86.6		13.4	0.03024	-
		2	151	76	97.3	95.8		4.2	0.05081	-
	M9G	1	160	35	97.1	67.6		32.4	0.09346	-
		2	116	78	67.9	0	84.9	15.1	0.05852	0

Movie S1 (separate file). Movies of cell growth, division, and death for example wt cells of phenotypes Ia, Ib, and II. The images are an overlay of DIC, the chromosome in green, and propidium iodide in red.

Movie S2 (separate file). An example of a cell that loses its PRJ2001-GFP-Fis plasmid and undergoes lysis due to the antibiotics present in the media. This cell loses its plasmid at 54:15 hr, proceeds to divide seven additional times, becomes filamented, and undergoes lysis at 56:15 hr.

References

1. Caspi, R. *et al.* The MetaCyc database of metabolic pathways and enzymes-a 2019 update. *Nucleic Acids Res* **48**, D455–D453 (2020).
2. Côté, J. P. *et al.* The genome-wide interaction network of nutrient stress genes in Escherichia coli. *mBio* **7**, (2016).
3. Edwards, J. S. & Palsson, B. O. *The Escherichia coli MG1655 in silico metabolic genotype: Its definition, characteristics, and capabilities*. vol. 97 http://gcrg.ucsd.edudownloads.html. (2000).
4. Bernhardt, T. G. & De Boer, P. A. J. SlmA, a nucleoid-associated, FtsZ binding protein required for blocking septal ring assembly over chromosomes in E. coli. *Mol Cell* **18**, 555–564 (2005).
5. Chen, J. C. & Beckwith, J. FtsQ, FtsL and FtsI require FtsK, but not FtsN, for co-localization with FtsZ during Escherichia coli cell division. *Mol Microbiol* **42**, 395–413 (2001).
6. Niki, H., Jaffe, A., Imamura, R., Ogura, T. & Hiraga, S. The new gene mukB codes for a 177 kd protein with coiled-coil domains involved in chromosome partitioning of E. coli. *EMBO Journal* **10**, 183–193 (1991).
7. Bendezú, F. O., Hale, C. A., Bernhardt, T. G. & De Boer, P. A. J. RodZ (YfgA) is required for proper assembly of the MreB actin cytoskeleton and cell shape in E. coli. *EMBO Journal* **28**, 193–204 (2009).
8. Pinto, C. & Shimakawa, K. A compressed logistic equation bacteria growth: Inferring time-dependent growth rate. *Phys Biol* (2022) doi:10.1088/1478-3975/ac8c15.
9. Tjørve, K. M. C. & Tjørve, E. The use of Gompertz models in growth analyses, and new Gompertz-model approach: An addition to the Unified-Richards family. *PLoS One* **12**, (2017).
10. Horowitz, J., Normand, M. D., Corradini, M. G. & Peleg, M. Probabilistic model of microbial cell growth, division, and mortality. *Appl Environ Microbiol* **76**, 230–242 (2010).
11. Yang, Y. *et al.* Temporal scaling of aging as an adaptive strategy of Escherichia coli. *Sci Adv* **5**, eaaw2069 (2019).
12. Takeuchi, R. *et al.* Colony-live - A high-throughput method for measuring microbial colony growth kinetics - Reveals diverse growth effects of gene knockouts in Escherichia coli. *BMC Microbiol* **14**, 1–11 (2014).
13. Guinn, L., Lo, E. & Balázsi, G. Drug-dependent growth curve reshaping reveals mechanisms of antifungal resistance in Saccharomyces cerevisiae. *Commun Biol* **5**, (2022).
14. Paintdakhi, A. *et al.* Oufti: An integrated software package for high-accuracy, high-throughput quantitative microscopy analysis. *Mol Microbiol* **99**, 767–777 (2016).
15. Baba, T. *et al.* Construction of Escherichia coli K-12 in-frame, single-gene knockout mutants: The Keio collection. *Mol Syst Biol* **2**, 2006–2008 (2006).
